# Ago-RIP Sequencing Identifies New MicroRNA-449a-5p Target Genes Increasing Sorafenib Efficacy in Hepatocellular Carcinoma

**DOI:** 10.7150/jca.66016

**Published:** 2022-01-01

**Authors:** Thea Reinkens, Amelie Stalke, Nicole Huge, Beate Vajen, Marlies Eilers, Vera Schäffer, Oliver Dittrich-Breiholz, Brigitte Schlegelberger, Thomas Illig, Britta Skawran

**Affiliations:** 1 Department of Human Genetics, Hannover Medical School, Hannover, Germany.; 2 Research Core Unit Genomics, Hannover Medical School, Hannover, Germany.; 3 Hannover Unified Biobank (HUB), Hannover Medical School, Hannover, Germany.

**Keywords:** Liver cancer, microRNA combination therapy, drug resistance, microRNA target genes, Ago-RIP sequencing, multi-tyrosine kinase inhibitor

## Abstract

**BACKGROUND:** Patients with hepatocellular carcinoma (HCC) have very limited treatment options. For the last fourteen years, the multi-tyrosine kinase inhibitor sorafenib has been used as standard-of-care therapeutic agent in advanced HCC. Unfortunately, drug resistance develops in many cases. Therefore, we aimed to find a way to mitigate drug resistance and to improve the sorafenib efficacy in HCC cells. MicroRNAs play a significant role in targeting genes involved in tumor control suggesting microRNA/sorafenib combination therapy as a promising treatment option in advanced HCC.

**METHODS:** MiR-449a-5p target genes were identified by Ago-RIP sequencing and validated by luciferase reporter assays and expression analyses*.* Target gene expression and survival data were analyzed in public HCC datasets. Tumor-relevant functional effects of miR-449a-5p and its target genes as well as their impact on the effects of sorafenib were analyzed using *in vitro* assays. An indirect transwell co-culture system was used to survey anti-angiogenic effects of miR-449a-5p.

**RESULTS:** *PEA15, PPP1CA* and* TUFT1* were identified as direct target genes of miR-449a-5p. Overexpression of these genes correlated with a poor outcome of HCC patients. Transfection with miR-449a-5p and repression of miR-449a-5p target genes inhibited cell proliferation and angiogenesis, induced apoptosis and reduced AKT and ERK signaling in HLE and Huh7 cells. Importantly, miR-449a-5p potentiated the efficacy of sorafenib in HCC cells via downregulation of *PEA15, PPP1CA* and* TUFT1.*

**CONCLUSIONS:** This study provides detailed insights into the targetome and regulatory network of miR-449a-5p. Our results demonstrate for the first time that targeting *PEA15, PPP1CA* and *TUFT1* via miR-449a overexpression could have significant implications in counteracting sorafenib resistance suggesting miR-449a-5p as a promising candidate for a microRNA/sorafenib combination therapy.

## Introduction

Hepatocellular carcinoma (HCC) is the fourth most common cause of cancer-related death worldwide, estimated to have been responsible for 781,631 deaths in 2018 1. While patients with early HCC benefit from potentially curative treatment options such as surgical resection or liver transplantation, patients with advanced HCC have a poor prognosis and very limited treatment options 2, 3. However, more than 80% of HCC patients are diagnosed at an advanced stage and are treated with the multi-tyrosine kinase inhibitor sorafenib as the first-line therapy 3, 4. Unfortunately, drug resistance in cancer therapy develops very often and is one of the main obstacles to overcome in curing this malignant disease 5, 6. According to statistical reports, more than 90% of deaths of tumor patients are associated with tumor drug resistance 7, 8. Therefore, a major goal of HCC research is to identify ways to mitigate drug resistance and to develop new strategies to improve the efficacy of therapeutic agents.

In recent years, it has become evident that the expression level of microRNAs (miRNAs) is globally reduced in cancer 9, 10. MicroRNAs are small non-coding RNAs involved in the posttranscriptional regulation of gene expression. By binding to complementary sites in the 3' untranslated regions (3'UTRs) of mRNAs they promote mRNA degradation or translational repression 11. MicroRNAs regulate hundreds of target genes and are powerful regulators of cell proliferation, apoptosis, cell migration and angiogenesis 12, 13. We have previously reported that miRNA expression profiles of HCC cell lines are regulated by epigenetic mechanisms and have demonstrated the tumor suppressive potential of miR-449a-5p 10, 14. Beyond this, significantly altered miRNA expression has been observed in a variety of drug-resistant HCC cells compared to drug-sensitive cells, suggesting that miRNAs may promote personalized HCC therapy 6,15. Consequently, microRNAs are a promising option in cancer therapy to increase drug efficacy and to improve patient outcome.

Sorafenib was the only available standard-of-care for advanced HCC for a decade 4. Currently, six systemic therapies have been FDA approved based on phase III trials (sorafenib, atezolizumab plus bevacizumab, lenvatinib, regorafenib, cabozantinib and ramucirumab) 4. Among them the application of multi-tyrosine kinase inhibitor sorafenib is still an effective first-line therapy exhibiting anti-angiogenic and antiproliferative effects 5, 16. Sorafenib suppresses tumor cell proliferation by inhibiting serine/threonine kinase Raf-1 in the RAF/MEK/ERK signaling pathway. In addition, sorafenib targets vascular endothelial growth factor receptor 2 (VEGFR) tyrosine kinase and other proteins to reduce tumor angiogenesis 17. However, only approximately 30% of patients can benefit from sorafenib and most of them acquire drug resistance within 6 months 5.

In the publication at hand, we focus on the characterization of the miR-449a-5p targetome and analyze the impact of miR-449a-5p and its target genes *PEA15, PPP1CA* and *TUFT1* on the efficacy of sorafenib in hepatocellular carcinoma.

## Materials and Methods

Detailed information is provided in the supplementary data.

### Ago2 RNA immunoprecipitation (Ago2-IP)

Ago2-IP was performed to identify direct miR-449a-5p target genes. 1 x 10^7^ HLE cells were transfected with miR-449a-5p or microRNA negative control. Lysed cell extracts were immunoprecipitated with Ago2 antibody (Chromotek, Planegg-Martinsried, Germany) or IgG isotype control (Chromotek) using Dynabeads Protein G (Invitrogen, Carlsbad, USA) and is described in detail in the supplementary data.

### Library generation and sequencing

Sequencing libraries were generated with the NEBNext Single Cell/ Low Input RNA Library Prep Kit for Illumina (New England Biolabs, Ipswich, USA). Enrichment and size distribution of the libraries were quality-assessed by Agilent Bioanalyzer 2100 on a DNA high sensitivity chip (Agilent, Santa Clara, USA). Three biological replicates were analyzed. Single-read sequencing was performed on an Illumina NextSeq 550 sequencer. Details are provided in the supplementary data.

### Statistical modeling and analysis of Ago-RIP sequencing (Ago-RIP-Seq)

The identification of direct miR-449a-5p targets was achieved by using the concept of linear contrasts 18. Following miR-449a-5p overexpression, the fraction of miR-449a-5p and its target genes is increased in the Ago complex. To reduce the noise due to unspecific RNA binding to the Protein G beads in the Ago-RIP-Seq experiment, the Ago-IP fractions were adjusted to IgG-IP [Contrast 1, C1] before comparing the Ago-IP fractions of miR-449a-5p with the Ago-IP of miR-control as follows:

[Contrast 1] = (Ago_miR-449a_ - IgG_miR-449a_) - (Ago_miR-Ctrl_ - IgG_miR-Ctrl_)

Since miR-449a-5p overexpression leads to widespread secondary changes within the gene expression profiles that might impact the profiles of immunoprecipitation 19, it was necessary to adjust the levels of RNAs detected in the Ago-IP fractions to their expression levels measured in the total lysates (=Input) [Contrast 2, C2] as follows:

[Contrast 2] = (Ago_miR-449a_ - Input_miR-449a_) - (Ago_miR-Ctrl_ - Input_miR-Ctrl_)

As microRNA targets are decreased after miR-449a-5p expression, the comparison [Contrast 3, C3] for identifying whole transcriptional changes was defined as follows:

[Contrast 3] = (Input_miR-449a_ - Input_miR-Ctrl_)

The contrasts C1, C2 and C3 define the type of comparison between factor levels. Wald-tests were applied for testing against zero.

### Analysis of mRNA, miRNA and protein expression

Total RNA was isolated using the Direct-zol RNA MiniPrep Kit (Zymo Research). 250 ng total RNA was transcribed into complementary DNA (cDNA) with the High-Capacity cDNA Reverse Transcription Kit (Thermo Fisher Scientific, Waltham, USA). Relative mRNA expression was measured in triplicate by quantitative real-time PCR using TaqMan Gene Expression Assays (Life Technologies) with *TBP* as reference gene. All TaqMan Assays used in this study are listed in the supplementary data.

For protein analysis, whole cell lysates were prepared with RIPA buffer and protein concentration was measured by Bradford protein assay. Equal amounts of protein were separated in a sodium dodecyl sulfate-polyacrylamide gel and protein levels were analyzed using a standard western blot protocol. Antibodies against PEA15, PPP1CA, TUFT1, (phosphor‑) AKT, (phospho-) ERK, α-actinin, β-actin and GAPDH were used (antibody dilutions are provided in the supplementary data).

### Luciferase reporter assay

To validate that *PEA15, PPP1CA* and *TUFT1* are direct target genes of miR-449a-5p, luciferase reporter assays were performed. For this, luciferase reporter vectors were constructed containing the 3'UTR of *PEA15, PPP1CA* or *TUFT1* either with intact miR-449a-5p binding sites or with mutated miR-449a-5p binding sites. Luciferase reporter assays were performed as described in the supplementary data.

### Assays to determine proliferation, apoptosis and angiogenesis

Cell viability and apoptosis were measured in triplicate every 24 h at four different times using WST-1 Proliferation Reagent (Roche) and the Caspase3/7 Glo Assay (Promega, Madison, USA), respectively. Angiogenesis was determined by performing a tube formation assay in an indirect co-culture system of HLE or Huh7 and HUVEC cells. Tube formation assays were performed as described in the supplementary methods.

### Analysis of public data sets

To analyze the expression of *PEA15, PPP1CA* and *TUFT1* in non-tumorous liver tissue and HCC tissue, expression levels of the NCBI GEO data sets GSE14520 and GSE22058 were downloaded (https://www.ncbi.nlm.nih.gov/geo/, 07.01.2021). In addition, clinical data of the TCGA-LIHC cohort were downloaded from the Cell Index Database CELLX 20. For survival analysis of the TCGA-LIHC cohort, survival data together with expression levels of *PEA15, PPP1CA* and *TUFT1* were downloaded from http://www.oncolnc.org/ (17.01.2021) and the expression was classified as high (upper median) or low (lower median).

### Statistics

Data are represented as mean ± standard deviation (SD) of at least three independent experiments. Statistical significance was determined with GraphPad Prism (GraphPad Software, Version 8) by two-tailed Student's t-tests, ordinary one-way ANOVA or by two-way ANOVA with Dunnett's or Sidaks multiple comparisons test. For experiments analyzing sorafenib-induced apoptosis and cell viability, statistical significance was determined by two-way ANOVA with Tukey's or Dunnett's multiple comparisons test as indicated.

## Results

### Ago-RIP sequencing identifies new miR-449a-5p target genes

**Figure 1:**
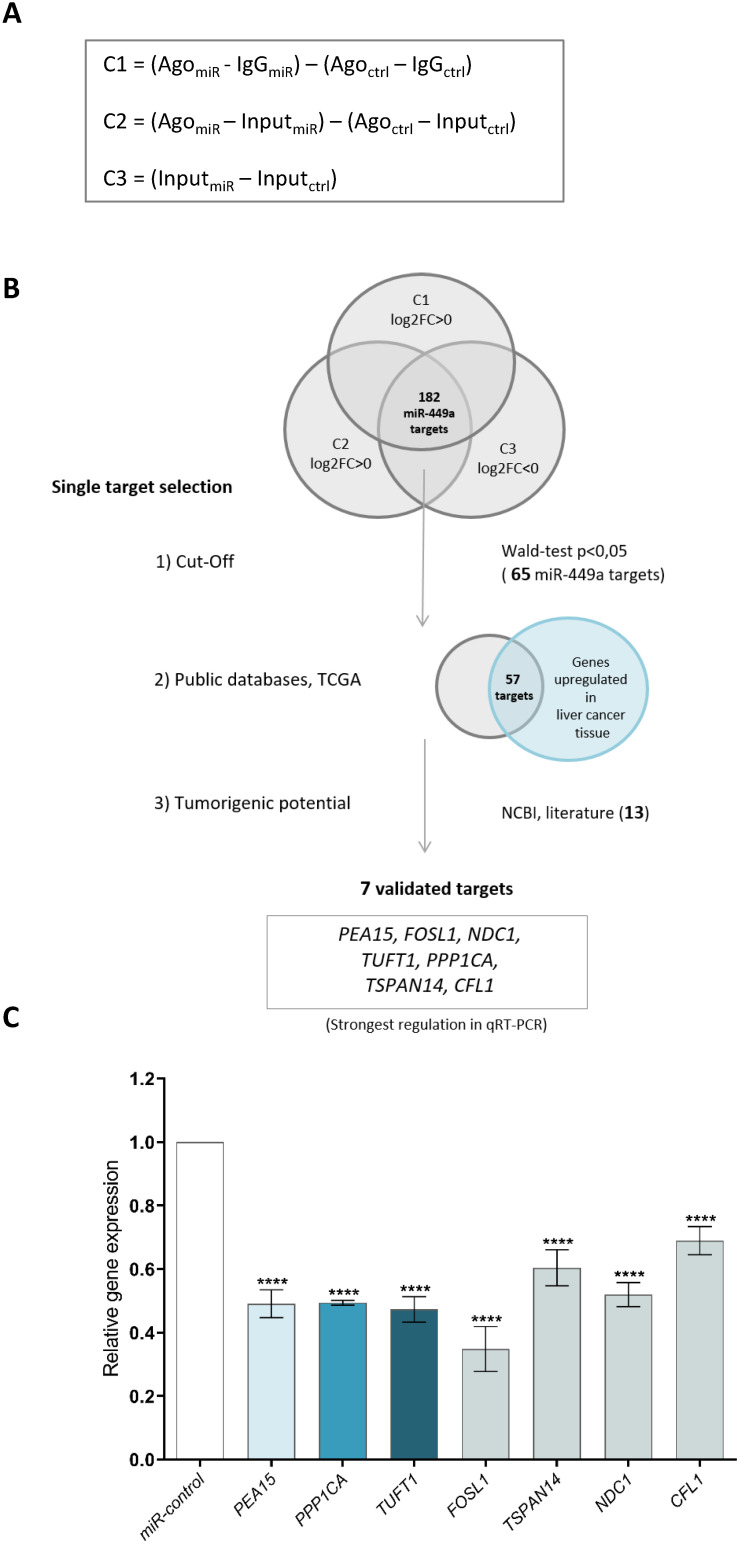
** Ago-RIP sequencing identifies new miR-449a-5p target genes.** (A) Comparisons calculated to detect potential direct miR-449a-5p target genes and transcriptional changes considering the concept of linear contrasts. (B) Workflow of miR-449a-5p target gene filtering and definition of potential direct miR-449a-5p targets. C = Contrast, FC = fold change. (C) Validation of miR-449a-5p targets in HLE cells by quantitative Real-Time PCR. Relative expression of target RNAs was normalized to miR-control treated HLE cells. ****p<0.0001; ordinary one-way ANOVA with Dunnett's multiple comparisons test.

Functional microRNAs are incorporated into the Ago-RISC complex and bind to the mRNAs of their target genes, which are subsequently translationally inhibited or degraded 21. To identify direct target genes of miR-449a-5p, we transfected HLE cells with miR-449a-5p mimics. After cell lysis (total lysate = input, input_miR_, input_ctrl_) we immunoprecipitated the Ago complexes from total lysates with Ago antibodies (Ago_miR_, Ago_ctrl_) and from controls with IgG antibodies (IgG_miR_, IgG_ctrl_) to remove unspecific background. The RNA of the input, Ago and IgG fractions of three independent Ago immunoprecipitation experiments were analyzed by high-throughput sequencing (Ago-RIP-Seq). To statistically evaluate the Ago-RIP-Seq results, three comparisons were established to identify direct miR-449a-5p target genes (Fig. 1A). Potential miR-449a target genes were defined by positive log2FC values in comparison 1 and comparison 2 and negative log2FC in comparison 3. Ago-RIP sequencing yielded 182 potential direct miR-449a-5p target genes that fulfilled these conditions in all three replicates (Fig. 1B, Supplementary Table 1). Target gene filtering was conducted to further prioritize the identified miR-449a targets for functional analyses (Fig. 1B). In a first cut-off 65 genes were selected with a Wald-test p-value smaller than 0.05. As an additional selection criterion, the expression of target genes was required to be higher in liver cancer tissue than in the adjacent liver tissue according to TCGA datasets. This prioritization yielded 57 direct miR-449a targets. As a result of manual literature search, 13 out of the 57 genes were identified as potential tumorigenic oncogenes not yet described as potential miR-449a-5p target genes. To validate these findings, we quantified the expression of seven genes (*PEA15, PPP1CA, TUFT1, FOSL1, TSPAN14, NDC1* and* CFL1*) by quantitative Real-Time PCR using input fractions of HLE miR-449a and HLE miR-control cells. 48 h after transfection mRNA expression levels were reduced in all seven cases (Fig. 1C). In the following, we focused on *PEA15, PPP1CA* and *TUFT1* because they showed a strong regulation in qRT-PCR and literature research of these genes indicated promising approaches for the treatment of hepatocellular carcinoma.

### *PEA15, PPP1CA* and *TUFT1* are direct target genes of miR-449a-5p

**Figure 2:**
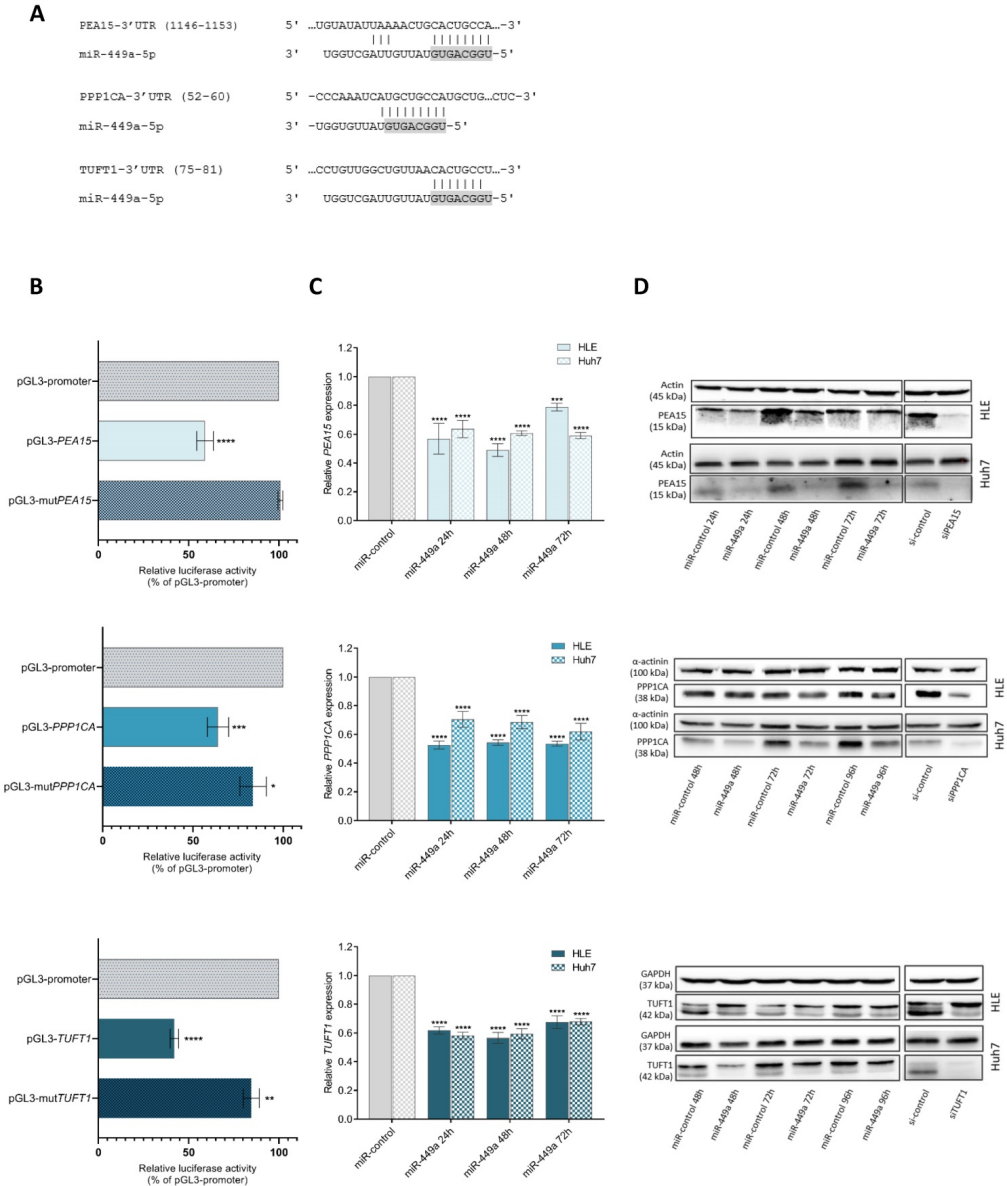
***PEA15, PPP1CA* and *TUFT1* are direct target genes of miR-449a-5p.** (A) Represented are predicted binding sites in the 3'UTR of *PEA15, PPP1CA* and *TUFT1* with the greatest binding energy for miR-449a-5p. Seed regions of miR-449a are highlighted in gray. (B) Firefly luciferase activity was measured and normalized to renilla luciferase activity. (C, D) RNA (C) and protein (D) expression of *PEA15, PPP1CA* and *TUFT1* was analyzed at three different times after transfection of HLE and Huh7 cells with miR-449a-5p or after transfection with siRNA pools. *p<0.05, **p<0.01, ***p<0.001, ****p<0.0001; two-way ANOVA with Dunnett's multiple comparisons test.

Next, we aimed to validate the predicted regulation of *PEA15, PPP1CA* and* TUFT1* by miR-449a-5p by performing luciferase reporter assays. For this, we used vectors harbouring the 3'UTRs of *PEA15, PPP1CA* or *TUFT1* including either intact or mutated binding sites for miR-449a-5p that were predicted by TargetScan 22 or IntaRNA 23. Fig. 2A shows the respective miR-449a-5p binding site with the strongest predicted binding energy for the interaction with *PEA15, PPP1CA* and *TUFT1* 3'UTR. Cotransfection of miR-449a with pGL3-wildtype 3'UTR of the candidate genes significantly reduced luciferase activity compared to the empty pGL3 promoter vector while there was no change upon cotransfection with pGL3-mut*PEA15* and only a minor reduction upon cotransfection with pGL3-mut*PPP1CA* and pGL3-mut*TUFT1* (Fig. 2B)*.* Furthermore, we analyzed the expression of *PEA15, PPP1CA* and *TUFT1* in HLE and Huh7 cells at different times after miR-449a-5p transfection. The mRNA levels of *PEA15, PPP1CA* and *TUFT1* were reduced by miR-449a-5p at all stages (Fig. 2C). This downregulation was also observed on protein levels (Fig. 2D). For functional analyses, siRNA pools against *PEA15, PPP1CA* and *TUFT1* were established (Suppl. Fig. S1). Western blot analyses after siPool transfection of HLE and Huh7 cells showed a reduced protein expression of *PEA15, PPP1CA* and *TUFT1* (Fig. 2D)*.* Together our results indicate that *PEA15, PPP1CA* and *TUFT1* are direct target genes of miR-449a-5p.

### Overexpression of *PEA15, PPP1CA* and *TUFT1* correlates with a poor survival prognosis of HCC patients

**Figure 3:**
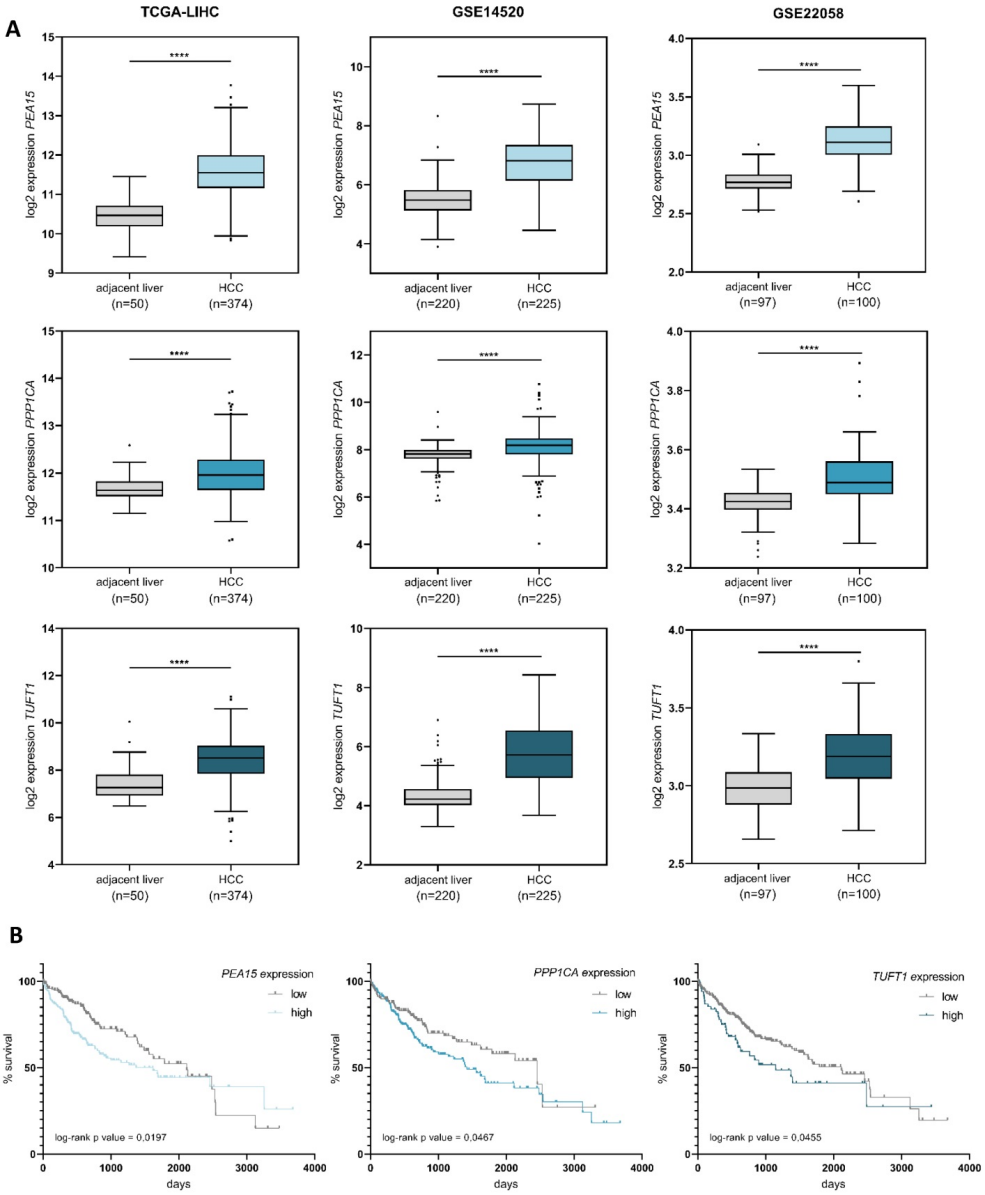
** Overexpression of *PEA15, PPP1CA* and *TUFT1* correlates with poor survival of HCC patients.** (A) Expression levels of *PEA15, PPP1CA* and *TUFT1* were analyzed using three public HCC data sets (TCGA-LIHC, GSE14520, GSE22058). *PEA15, PPP1CA* and *TUFT1* expression was significantly higher in HCC tissue than in adjacent non-tumorous liver tissue. Tukey box-and-whisker plot. ****p<0.0001; two-tailed Student's t test (B) Expression values of *PEA15, PPP1CA* and *TUFT1* and survival data of the TCGA-LIHC cohort were retrieved from OncoLnc 51. Patients were grouped into low or high mRNA expression levels with different percentiles (*PEA15* = 50th percentile, *PPP1CA* = 40th percentile, *TUFT1* = 80th percentile). Kaplan-Meier with log-rank test.

To determine the relevance of *PEA15, PPP1CA* and *TUFT1* in hepatocellular carcinoma *in vivo*, we analyzed three publically available expression datasets of primary HCCs. In all three datasets the expression levels of *PEA15, PPP1CA* and *TUFT1* were significantly increased in liver cancer tissue compared to adjacent non-tumorous liver tissue (Fig. 3A). Furthermore, we observed that patients with high expression of *PEA15, PPP1CA* or *TUFT1* had a trend towards an unfavourable survival prognosis (Fig. 3B). Our findings demonstrate that *PEA15, PPP1CA* and *TUFT1* are frequently overexpressed in HCC and that patients with hepatocellular carcinoma may benefit from the repression of these genes.

### Knockdown of *PEA15, PPP1CA* and *TUFT1* exerts distinct tumor suppressive functions

**Figure 4:**
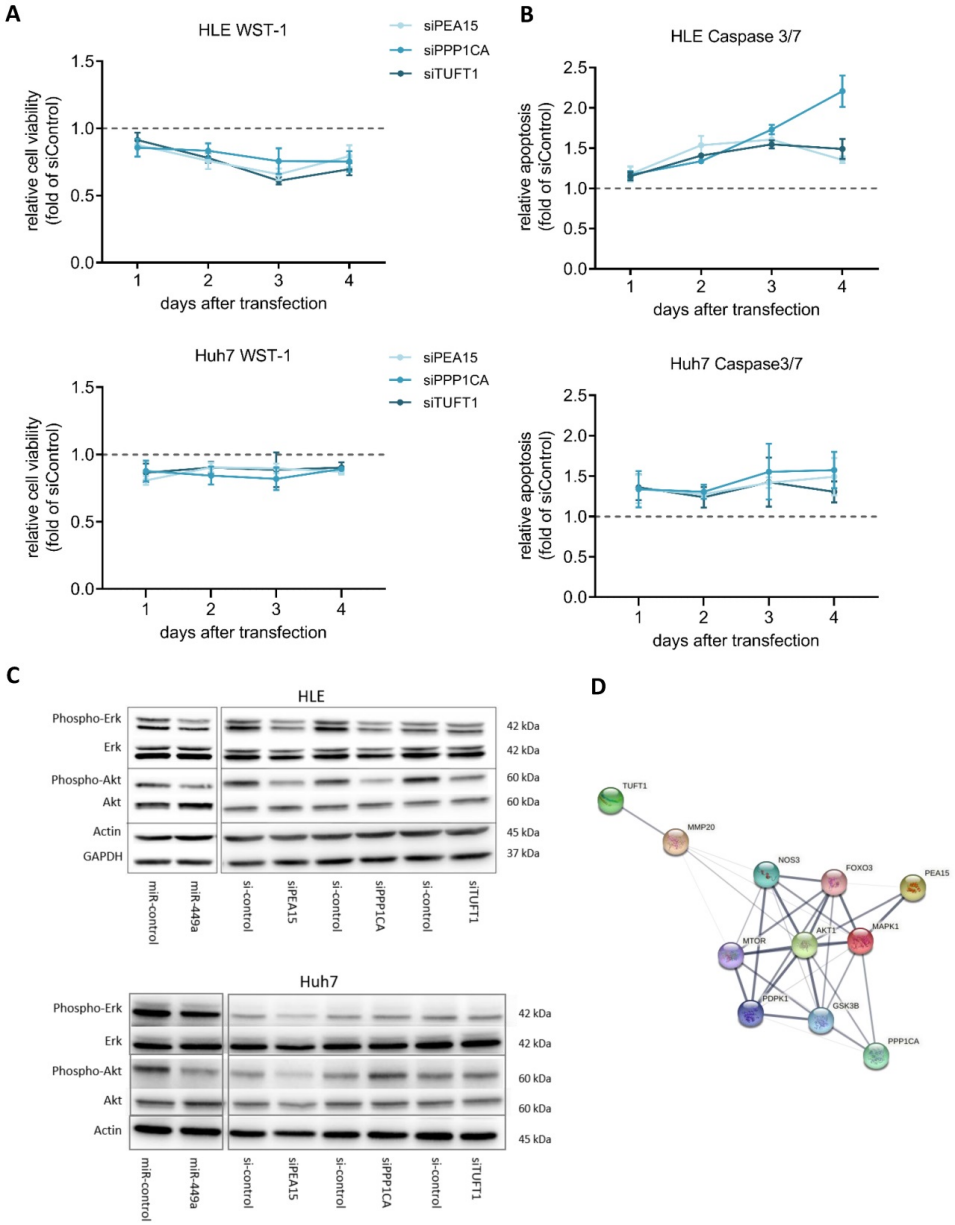
** miR-449a and knockdown of *PEA15, PPP1CA* and *TUFT1* exert distinct tumor suppressive functions.** (A,B) HLE and Huh7 cells were transfected with 3 nM siRNA pools against the expression of either *PEA15, PPP1CA* or *TUFT1*. (A) Cell viability was analyzed by WST-1 assay and normalized to si-control (dotted line). (B) Apoptosis was analyzed by caspase 3/7 assay and normalized to cell viability and si-control (dotted line) (C) HLE and Huh7 cells were transfected with miR-449a-5p mimic or siRNA pools against the expression of either *PEA15, PPP1CA* or *TUFT1*. 48 h after transfection, protein expression of p-ERK 1/2, ERK 1/2, p-Akt and Akt was analyzed by western blotting with Actin and GAPDH as loading controls. Gels were processed in parallel. (D) Protein association network in STRING illustrating the interaction with MAPK1 (ERK) and AKT1. Line thickness indicates the strength of data support 25.

Next, we analyzed the functional effects of *PEA15, PPP1CA* and *TUFT1* by performing cell viability and apoptosis measurements. For this, HCC cell lines HLE and Huh7 were transiently transfected using specific siRNA pools against *PEA15, PPP1CA* and *TUFT1*. Knockdown of *PEA15, PPP1CA* and *TUFT1* decreased cell viability (Fig. 4A) and increased apoptosis (Fig. 4B) in each case, whereby knockdown of *PPP1CA* showed the strongest induction of apoptosis. To further clarify the influence of miR-449a-5p and its target genes *PEA15, PPP1CA* and *TUFT1* on the regulation of HCC related pathways, western blot analyses were performed (Fig. 4C). AKT and ERK 1/2 are active in their phosphorylated state enhancing growth and survival of hepatocellular carcinoma 24. In this study, transfection of miR-449a-5p led to a reduced phosphorylation of AKT and ERK 1/2 in HLE cells, whereas the total concentration of AKT and ERK 1/2 remained unchanged. In addition, phosphorylation of AKT and ERK 1/2 was also decreased after siRNA induced knockdown of *PEA15, PPP1CA* and *TUFT1* with the exception that downregulation of *TUFT1* only reduced AKT phosphorylation (Fig. 4C). In Huh7 cells, miR-449a overexpression decreased AKT phosphorylation and the knockdown of *PEA15* led to a reduced AKT and ERK signalling (Fig. 4C). A STRING functional protein association network 25 confirmed the interaction of PEA15, PPP1CA and TUFT1 with AKT and ERK (MAPK1) whereby PEA15 indicated the strongest interaction (Fig. 4D). In summary, knockdown of all three validated miR-449a target genes exerted tumor suppressive functions and has an impact on AKT and ERK signaling, although the extent of the observed effects differed.

### miR-449a-5p overexpression and the repression of its target genes *PEA15, PPP1CA* and *TUFT1* prevent tumor angiogenesis

**Figure 5:**
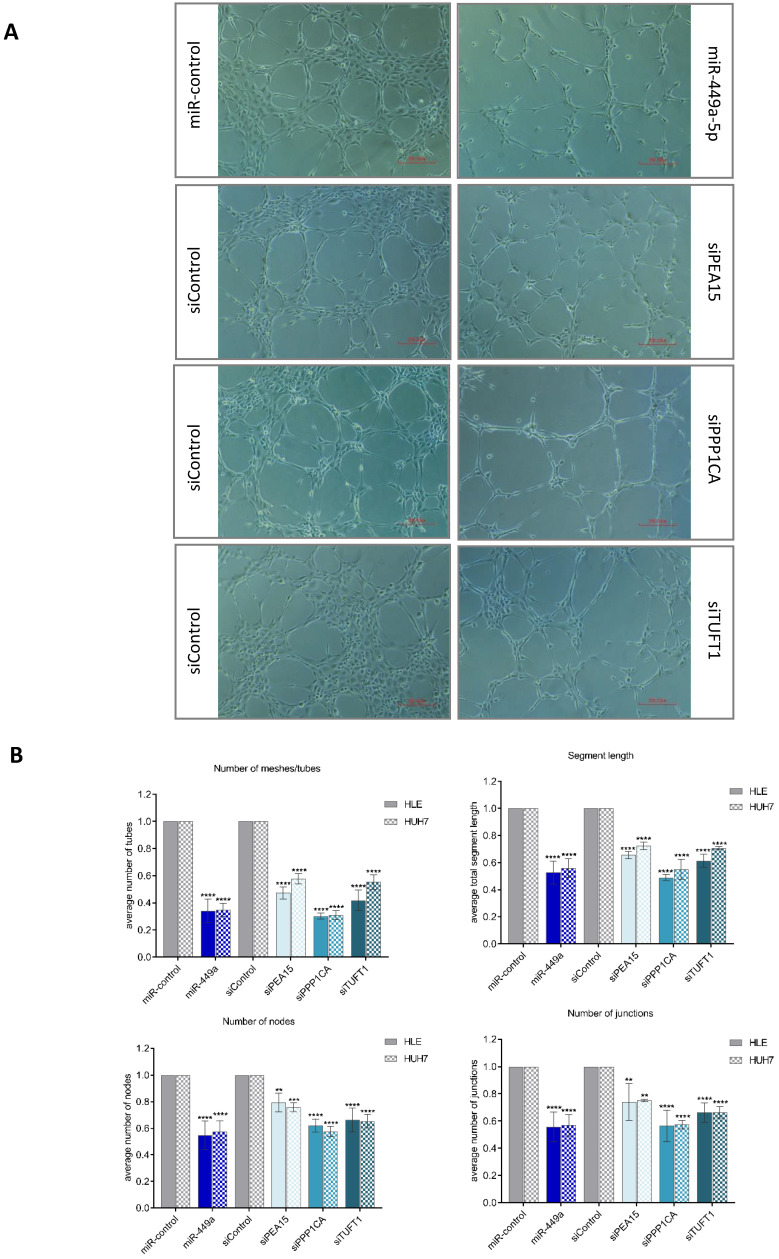
** miR-449a-5p and knockdown of *PEA15, PPP1CA* and *TUFT1* reduce endothelial cell tube formation.** (A) Representative images of capillary-like tubular structures of human umbilical vein endothelial cells (HUVEC) on Matrigel when co-cultured with miR-449a-5p/miR-control or siRNA/si-control transfected HLE or Huh7 cells. Angiogenic changes were imaged using an inverted light microscope. Scale bar = 200.00 µm (B) Angiogenic parameters (number of tubes, segment length, number of junctions and number of nodes) were quantified with Angiogenesis Analyzer from ImageJ. *p<0.05, **p<0.01, ***p<0.001, ****p<0.0001; two-way ANOVA with Sidak's multiple comparisons test.

To investigate the impact of miR-449a-5p and its target genes on tumor angiogenesis, we established a co-culture system with endothelial cells (HUVECs) using a 0.4 µm polycarbonate membrane. We used phenotypic changes of HUVECs to evaluate the effects of transiently transfected liver cancer cells on angiogenesis in the co-culture system. HUVECs, co-cultured with HLE or Huh7 cells that were transfected with a negative control, formed a tight cluster of capillary-like tubes and created a mesh-like structure on matrigel. In contrast, after 6 h of co-culture with miR-449a-5p transfected cells, HUVECs elongated, lost contact with each other and the capillary-like tubular structure was less dense, as shown in Fig. 5A. HLE and Huh7 cells transfected with siRNA pools against *PEA15, PPP1CA* or *TUFT1* caused similar morphological changes of HUVECs, whereby transfection of siPPP1CA indicated the strongest anti-angiogenic impact. Quantitative analysis of capillary-like tubular structures confirmed the visual results. Quantification showed that the average number of tubes, junctions and nodes as well as the total segment length were significantly decreased when HUVECs were co-cultured with miR-449a or siRNA transfected HLE cells (Fig. 5B). An indirect co-culture of HUVECs with transfected Huh7 cells showed similar results. As observed in microscopic examination, transfection of miR-449a-5p as well as knockdown of *PPP1CA* quantitatively caused the most anti-angiogenic effects (Fig. 5B). Altogether, our results provide evidence for the prevention of tumor angiogenesis through the repression of *PEA15, PPP1CA* and *TUFT1* demonstrating the importance of their downregulation by miR-449a-5p.

### miR-449a-5p increases sorafenib efficacy of hepatocellular carcinoma cells via downregulation of *PEA15, PPP1CA* and *TUFT1*

**Figure 6:**
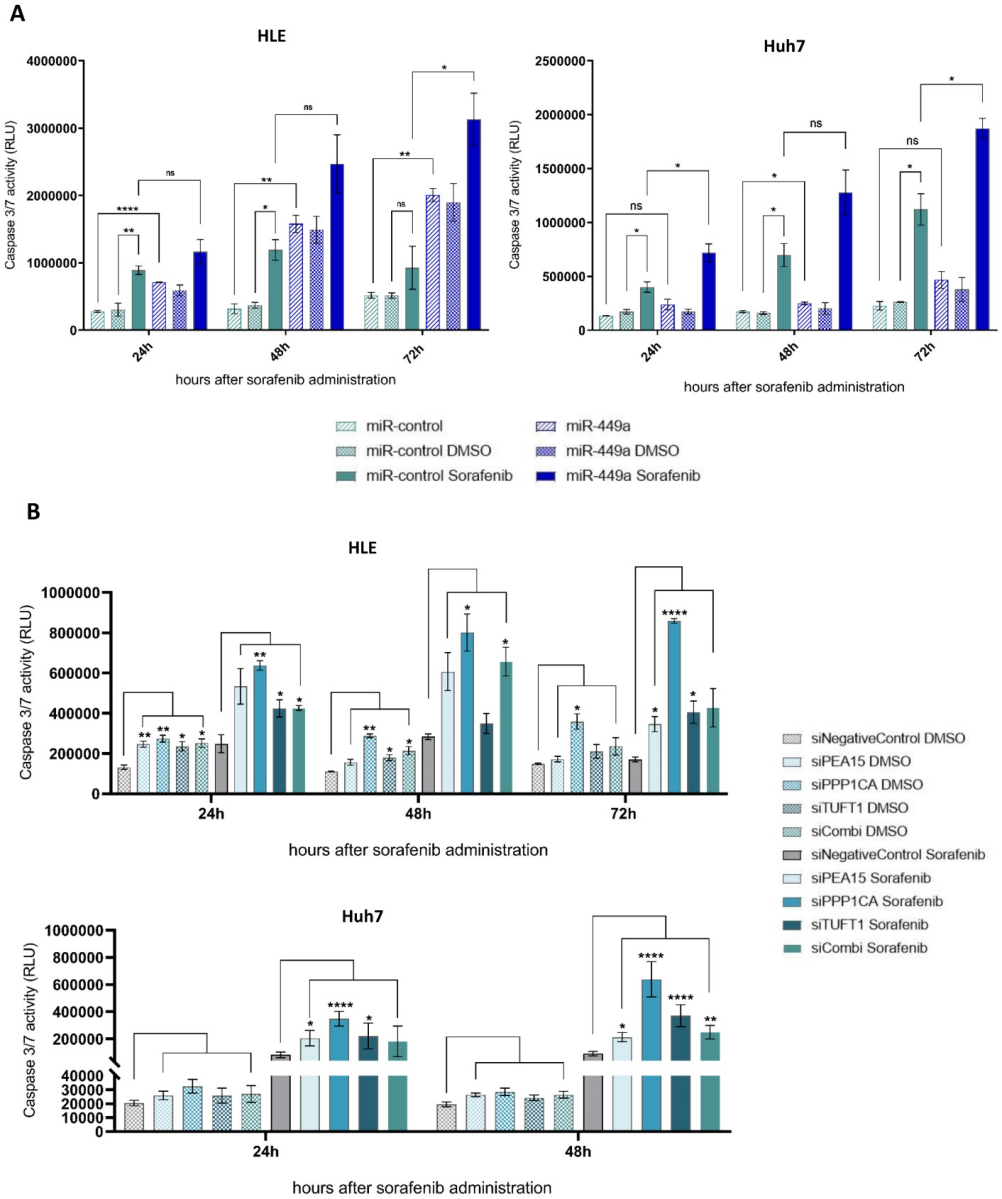
** miR-449a-5p and knockdown of *PEA15, PPP1CA* and *TUFT1* increase sorafenib efficacy of hepatocellular carcinoma cells.** (A) HLE and Huh7 cells were transfected with miR-449a-5p mimic/ miR-control and treated with 10 µM sorafenib or DMSO vehicle control. 24 h, 48 h and 72 h after sorafenib treatment tumor cell apoptosis and cell viability were measured. Apoptosis was normalized to cell viability (Suppl. Fig. S2A); two-way ANOVA with Tukey's multiple comparisons (square brackets). (B) HLE and Huh7 cells were transfected with siRNA pools, either alone or in combination (siCombi) and treated with 10 µM sorafenib or DMSO vehicle control. 24 h, 48 h and 72 h after sorafenib treatment tumor cell apoptosis and cell viability were measured. Apoptosis was normalized to cell viability (Suppl. Fig. S2B); *p<0.05, **p<0.01, ***p<0.001, ****p<0.0001; two-way ANOVA with Dunnett's multiple comparisons test (square brackets). RLU = relative light units.

The fact that an increased expression of angiogenesis-related genes as well as an upregulation of MAPK/ERK signaling is associated with sorafenib resistance 26, 27 led us to investigate the impact of miR-449a-5p and its target genes on the effect of sorafenib in hepatocellular carcinoma. First, cell viability and apoptosis of HLE and Huh7 cells were measured at different times after miR-449a-5p overexpression and additional sorafenib treatment. HCC cells transfected with miR-449a-5p and treated with sorafenib exhibited an increased apoptosis at all times compared to cells only treated with sorafenib (Fig. 6A). 72 hours after sorafenib administration, miR-449a-5p significantly tripled sorafenib-induced apoptosis of HLE cells and significantly doubled sorafenib-induced apoptosis of Huh7 cells. Although miR-449a-5p did not significantly enhance the antiproliferative effects of sorafenib, examination of cell viability demonstrated less absorbance in case of HCC cells additionally transfected with miR-449a-5p compared to HCC cells only treated with sorafenib (Suppl. Fig. S2A). Since previous results indicated that knockdown of *PEA15, PPP1CA* and *TUFT1* by miR-449a-5p exerts tumor suppressive functions, we further analyzed the impact of miR-449a-5p target genes on sorafenib efficacy. Therefore, we transiently transfected HLE and Huh7 cells with siRNAs against *PEA15, PPP1CA* and *TUFT1* either alone or in combination (siCombi) and investigated apoptosis and cell viability after sorafenib treatment or DMSO vehicle control administration (Fig. 6B). In total, the apoptosis of HCC cells treated with sorafenib was stronger compared to HCC cells without sorafenib administration at all times. Furthermore, siRNA-mediated knockdown of *PEA15, PPP1CA* and *TUFT1* greatly enhanced the apoptotic effects of sorafenib, whereby siPPP1CA showed the most significant impact (Fig. 6B). Cell viability reflected these results and demonstrated a significant decrease of cell proliferation after additional knockdown of *PEA15, PPP1CA* and *TUFT1* compared to single sorafenib treatment (Suppl. Fig. S2B). Here again, downregulation of *PPP1CA* caused the most antiproliferative impact. Altogether, the results indicate that miR-449a-5p sensitized hepatocellular carcinoma cells to sorafenib by downregulation of *PEA15, PPP1CA* and *TUFT1,* whereby knockdown of *PPP1CA* had the greatest influence. On top of that, the observed impacts of miR-449a-5p and its target genes on the effects of sorafenib-mediated apoptosis were indicative for the strong tumor suppressive capability of miR-449a and its promising potential in a combination therapy with sorafenib.

## Discussion

In this study, we comprehensively investigated the impact of miR-449a-5p and its target genes *PEA15, PPP1CA* and *TUFT1* on the effects of sorafenib treatment in hepatocellular carcinoma. Specifically, we examined whether miR-449a-5p is a suitable candidate for a microRNA/ sorafenib combination therapy. Several studies have reported that microRNAs play an emerging role in drug resistance of hepatocellular carcinoma 15, 28, 29 suggesting that a microRNA/ sorafenib combination therapy is an attractive option for HCC therapy. Here, we demonstrated that miR-449a-5p strongly potentiates sorafenib-induced apoptosis of hepatocellular carcinoma cells by downregulating *PPP1CA* in particular.

As we have previously shown, miR-449a and several other microRNAs are epigenetically deregulated in HCC, so that the role of microRNAs in tumorigenesis has come into focus 10. In addition, we have demonstrated that miR-449a inhibits proliferation and induces apoptosis *in vitro* and suppresses tumor growth *in vivo* in hepatocellular carcinoma 10, 14. Similar tumor suppressive effects of miR-449a have also been observed in breast 30, lung 31, gastric 32 and prostate cancer 33. In this study, we confirmed the tumor suppressive potential of miR-449a-5p and demonstrated its anti-angiogenic effect. We further deciphered the large network of genes and signaling pathways that are regulated by miR-449a-5p by Ago-RIP sequencing and, thereby, contributed to the knowledge on its tumor suppressive potential. Seven significantly downregulated mRNAs were detected and showed a strong regulation in qRT-PCR. Literature research led us to focus our study on *PEA15, PPP1CA* and *TUFT1* since they represent promising targets for the treatment of HCC. This is the first study that deciphered the miR-449a-5p targetome via Ago-RIP sequencing and that demonstrated a negative impact of miR-449a-5p on angiogenesis in hepatocellular carcinoma.

Our study provides a link between *PEA15, PPP1CA, TUFT1* and miR-449a-5p. Performing luciferase reporter assays and western blottings, we demonstrated that *PEA15, PPP1CA* and *TUFT1* are direct target genes of miR-449a. We revealed overexpression of *PEA15, PPP1CA* and *TUFT1* in three different gene expression datasets of HCC providing evidence for an oncogenic role of these genes in HCC. Other studies have likewise reported an upregulation of *PEA15* 26 and *TUFT1* 34 in HCC tissues. In addition, we showed that HCC patients with high levels of *PEA15, PPP1CA* or *TUFT1* have a shorter survival than patients with a low expression of these genes. Overexpression of *PEA15, PPP1CA* and *TUFT1* and a related poorer outcome have also been observed in other tumor entities 35-37. Taken together, *PEA15, PPP1CA* and *TUFT1* appear to act as oncogenes and, therefore, their inhibition or downregulation may improve HCC therapy.

**Figure 7:**
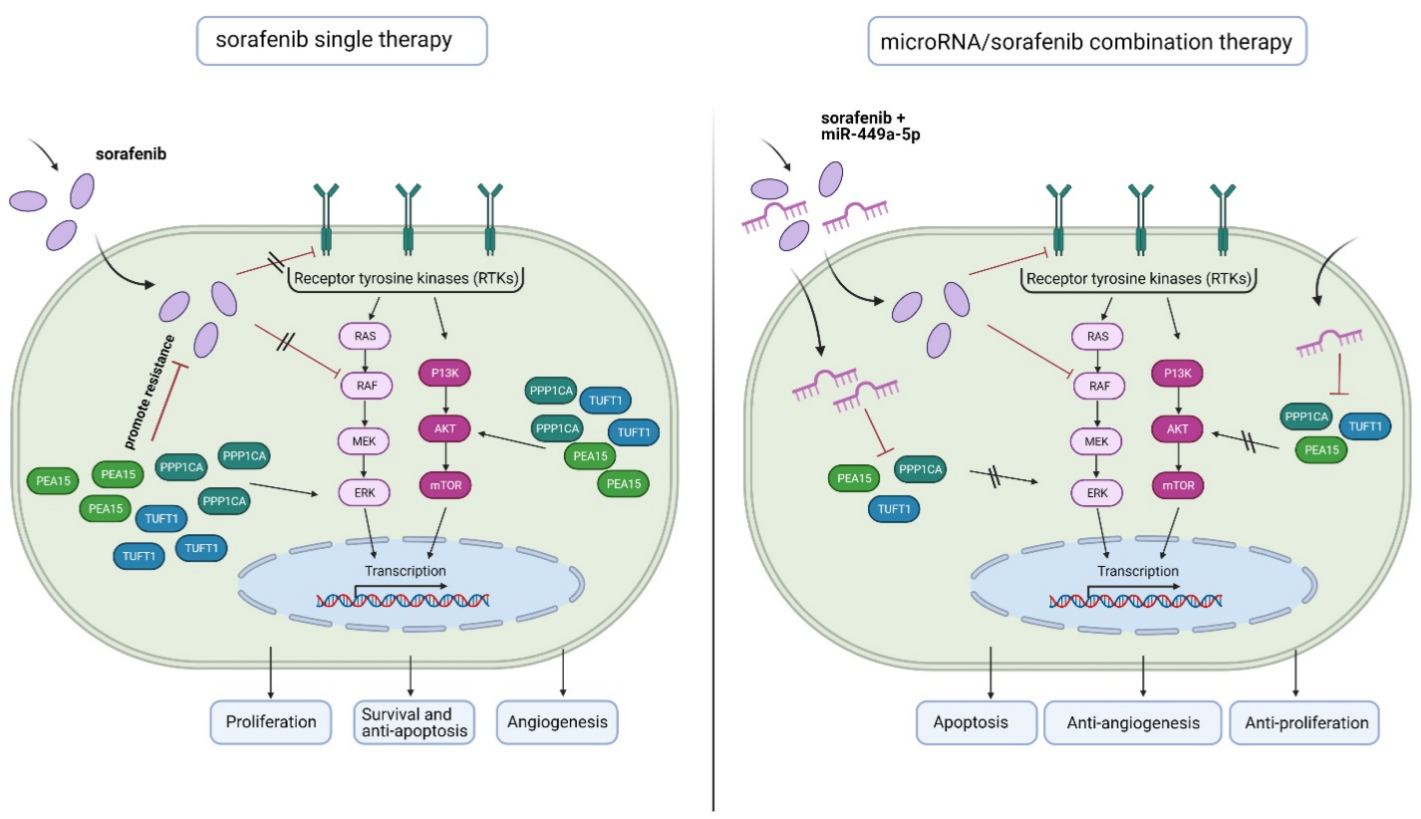
** Schematic diagram depicting the benefit of microRNA/ sorafenib combination therapy.** (Left) Sorafenib resistance in hepatocellular carcinoma is associated with an increased expression of angiogenesis and hypoxia-related genes (*TUFT1*) as well as an upregulation of ERK and AKT signaling (*PEA15, PPP1CA*). (Right) A microRNA-449a/ sorafenib combination therapy increases sorafenib efficacy via downregulation of miR-449a target genes *PPP1CA, PEA15* and *TUFT1.* This downregulation results in an increased apoptosis and a decreased angiogenesis and tumor cell proliferation. Created with BioRender.com.

Possible roles in tumorigenesis are already known for each of the three examined genes. *PEA15* encodes a death effector domain-containing phosphoprotein that functions as a negative regulator of apoptosis 38 and has been described to be involved in cell proliferation, migration and upregulating the ERK/MAPK signaling pathway 26,39. However, *PEA15* has a dual role in tumor regulation depending on its phosphorylation status and the cellular environment 39, 40. It has been revealed that phosphorylation of PEA15 has promoted the proliferation and invasion of gastric cancer cells via ERK phosphorylation 35, whereas the unphosphorylated state has inhibited the ERK and EGFR phosphorylation, thus inhibiting proliferation, invasion and metastasis of breast and ovarian cancer 39. *PPP1CA* encodes the subunit of serine/threonine specific phosphatase PP1 and is involved in multiple cellular functions including proliferation, invasion, cell survival and differentiation 41, 42. As a B-Raf activating phosphatase it also plays a role in the upregulation of ERK/MAPK signaling 43, 44. *TUFT1* encodes an acidic protein that is involved in angiogenesis, in the adaptation of hypoxia and promotes tumor growth and metastasis of HCC by activating the PI3K/AKT pathway 34, 45, 46. Interestingly, many studies have provided evidence that an increased expression of angiogenesis and hypoxia-related genes as well as an upregulation of ERK and AKT signaling is also associated with sorafenib resistance in hepatocellular carcinoma (Fig. 7) 5, 8, 26, 27. In addition, it is already known that *PEA15* confers resistance to sorafenib in HCC 26.

In line with these observations, we showed that knockdown of *PEA15, PPP1CA* and* TUFT1* increases apoptosis, decreases cell viability and significantly reduces angiogenesis in HLE and Huh7 cells. In addition, downregulation of *PEA15, PPP1CA* and* TUFT1* decreased AKT signaling and knockdown of *PEA15* and *TUFT1* also reduced ERK signaling. These results indicate that miR-449a-5p and its target genes play a role in counteracting sorafenib resistance (Fig. 7). Consistent with our findings, we demonstrated that miR-449a-5p overexpression as well as downregulation of *PEA15, PPP1CA* and *TUFT1* significantly potentiated sorafenib-induced apoptosis, whereby knockdown of *PPP1CA* had the greatest impact. Beyond this, Wei et al. have observed significantly altered miRNA expression in a variety of drug-resistant HCC cells, compared to those in drug-sensitive cells, suggesting that miRNAs may promote individualized HCC therapy 15. In this study, miR-449a-5p enhanced the efficacy of sorafenib via silencing *PEA15, PPP1CA* and *TUFT1.* These results are in line with Yang et al. who have reported that the miR-449a-5p related miR-34 reduces cell viability, promotes cell apoptosis and enhances sorafenib-induced apoptosis in HCC cell lines 47. Thus, our data suggest that targeting *PEA15, PPP1CA* and *TUFT1* by miR-449a-5p overexpression may have significant implications in potentiating the effects of sorafenib therapy.

Various studies have strongly suggested that counterbalancing the expression of microRNAs in drug resistant cells can re-sensitize cancer cells to therapeutic agents 29. However, there is no clinical trial for microRNA/sorafenib combination therapy yet. For further development of microRNA therapies, an extensive characterization of candidate microRNAs and detailed knowledge of their specific target, regulated pathways and functional effects is essential 14. Accordingly, we analyzed the miR-449a-5p targetome and its effect on sorafenib efficacy in HCC.

Recently, immunotherapy (atezolizumab plus bevazizumab) has been FDA approved as additional first-line therapy in advanced HCC 4. Even though immunotherapy is a promising therapy option for advanced HCC, it can unbalance the immune system and result in a wide range of immune-related adverse events 48. Studies have shown that HCCs belonging to the immune-excluded class, characterized by Wnt/ß-Catenin mutations, are proposed to be primarily resistant to immunotherapy 4, 49. Furthermore, Pfister et al. recently revealed that immunotherapy did even impair overall survival in patients with non-viral HCC 50. That is why sorafenib continues to be an important first-line therapy option especially in non-viral related HCC. The combination of sorafenib with miR-449a may extend the overall survival of HCC patients and reactivates sorafenib's attractiveness in advanced hepatocellular carcinoma.

## Conclusions

In conclusion, we here present the first evidence that miR-449a-5p increases the efficacy of sorafenib in HCC cells. Furthermore, our data suggest that counterbalancing microRNA expression may have significant implications in potentiating the effect of sorafenib therapy. Although further validation is required before miR-449a-5p may enter the routine clinical scenario. Our study provides evidence that miR-449a-5p is a promising candidate for a microRNA/sorafenib combination therapy. Therefore, miR-449a-5p may be a novel option for the treatment of patients with advanced HCC.

## Supplementary Material

Supplementary figures and tables.

## Abbreviations

Ago-RIP: Argonaute-2-RNA-immunoprecipitation
AKT: AKT Serine/Threonine Kinase 1
C1/C2/C3: Contrast 1/2/3
ERK: extracellular-signal regulated kinases
FDA: Food and Drug administration
HCC: Hepatocellular carcinoma
MEK: Mitogen-activated protein kinase kinase
miR-449a: microRNA-449a-5p
miRNA/miR: microRNA
PEA15: Proliferation And Apoptosis Adaptor Protein 15
PPP1CA: Protein Phosphatase 1 Catalytic Subunit Alpha
Raf-1: Raf-1 Serine/Threonine Kinase
RISC: RNA-induced silencing complex
RLU: relative light units
Seq: Sequencing
TUFT1: Tuftelin 1
VEGFR: Vascular Endothelial Growth Factor Receptor
3'UTR: 3' untranslated region
