# Supplementary material for: Ago-RIP Sequencing Identifies New MicroRNA-449a-5p Target Genes Increasing Sorafenib Efficacy in Hepatocellular Carcinoma
Source: J Cancer. 2022 Jan 1;13(1):62–75. doi: 10.7150/jca.66016 (PMC8692677; doi:10.7150/jca.66016)
Supplement: Supplementary file 1 — Supplementary figures and tables. [file jcav13p0062s1.pdf]

Supplementary data

Ago-RIP Sequencing Identifies New MicroRNA-449a-5p Target Genes Increasing Sorafenib Sensitivity in Hepatocellular Carcinoma

Thea Reinkens, Amelie Stalke, Nicole Huge, Beate Vajen, Marlies Eilers, Vera Schäffer, Oliver Dittrich-Breiholz, Brigitte Schlegelberger, Thomas Illig, Britta Skawran[1]

[1] **Corresponding author,** Department of Human Genetics, Hannover Medical School, Carl-Neuberg-Straße 1, 30625 Hannover, Germany, email: Skawran.britta@mh-hannover.de

Supplementary materials and methods 2
- Cell culture and transfection 2
- MiRNA mimics and siRNA Pools for transfection 2
- Library generation, sequencing, and raw data processing 3
  - Library generation, quality control, and quantification: 3
  - Library denaturation and Sequencing run: 3
  - BCL to FASTQ conversion: 3
  - Raw data processing and quality control: 4
  - Normalization and relative RNA enrichment analysis: 4
- Luciferase reporter assay 4
- Tube formation assay 5
- Taqman Assays for quantitative real-time PCR 5
- Antibodies for western blotting 6
- Antibodies for Ago2 RNA immunoprecipitation. 7
- Buffer for Ago2 RNA immunoprecipitation: 7
- gblocks for cloning luciferase reporter vectors 8

Supplementary Figures 13
- Suppl. Fig. 1. siPOOL establishment of siPEA15, siPPP1CA and siTUFT1 13
- Suppl. Fig. 2. MiR-449a-5p and knockdown of *PEA15, PPP1CA* and *TUFT1* increase sorafenib efficacy in HCC 15

Supplementary Tables 16
- Supplementary Table 1. Potential miR-449a-5p target genes identified by Ago-RIP-Seq 16

References 18

Supplementary materials and methods

Cell culture and transfection

HCC cell lines, HLE and Huh7, were kindly provided by Professor Nam-Ho Huh (Department of Cell Biology, Graduate School of Medicine, Dentistry and Pharmaceutical Sciences, Okayama University, Okayama, Japan) and cultured in Dulbecco's Modified Eagle Medium (DMEM) containing 10% FCS, 2mM L-glutamine and 100 U/mL penicillin/streptomycin. Human umbilical vein endothelial cells (HUVECs, pooled donors) were obtained from Lonza and cultured in Endothelial Basal Medium-2 (EBM-2) with supplements (Lonza, Basel, Switzerland). Human embryonic kidney cells (HEK293T) were cultured in DMEM containing 10% FCS, 100 U/mL penicillin/streptomycin, 3,7 g/l sodium hydrogen carbonate, 3,7 g/l D-Glucose und 1 mM sodium pyruvate. All cell lines were cultured at 37°C and 5 % CO_2 in a humidified incubator. Cells were transfected with 50 nM miR-449a-5p mimics (Qiagen) or 3 nM siPOOLs (siTOOLs Biotech) against *PEA15, PPP1CA* or *TUFT1* using HiPerFect Transfection Reagent (Qiagen). Allstars Negative Control (Qiagen) was used as non-targeting miR-control or siRNA-control. SiPOOLs against *PEA15, PPP1CA* or *TUFT1* were either single transfected (3 nM) or co-transfected in equimolar amounts. The medium was changed 24 h after transfection. For analysis of sorafenib-induced apoptosis, cells were treated with 10 µM sorafenib [1] or DMSO vehicle control 24h after transfection.

MiRNA mimics and siRNA Pools for transfection*.*

Oligonucleotide	Order no./ ID	Manufacturer
Allstars Negative Control	SI03650318	Qiagen, Hilden, Germany
Syn-hsa-miR-449a	MSY0001541	Qiagen, Hilden, Germany
siPOOL Negative Control	siPOOL Negative Control	siTOOLs Biotech, Martinsried, Germany
siPOOL PEA15	siPOOL 8682 PEA15 human	siTOOLs Biotech, Martinsried, Germany
siPOOL PPP1CA	siPOOL 5499 PPP1CA human	siTOOLs Biotech, Martinsried, Germany
siPOOL TUFT1	siPOOL 7286 TUFT1 human	siTOOLs Biotech, Martinsried, Germany

Ago2 RNA immunoprecipitation (Ago2-IP)

For Ago2-IP, miR-449a-5p or microRNA negative control transfected HLE cells (1 x 10^7) were lysed in 3x NP40 lysis buffer, incubated for 20 min on ice and centrifuged (15 min, 13.000 x g, 4 °C). 20 µl of the supernatant were stored for the input control. Dynabeads Protein G (Invitrogen, Carlsbad, USA) were prepared by 3 x washing with citrate buffer. Afterwards, 10 µg Ago2 antibody (Chromotek, Planegg-Martinsried, Germany) or 10 µg IgG isotype control (Chromotek) were coupled to the beads for 1 h at 4 °C. IP of the lysates was performed for 1 h rotating at 4 °C followed by three washing steps with IP wash buffer and high salt buffer and

two washing steps with phosphatase wash buffer and PNK buffer. RNA was isolated using 350 µl TRI Reagent (Zymo Research, Freiburg, Germany) followed by the Direct-zol RNA MiniPrep Kit (Zymo Research). As quality controls, microRNA levels were measured in the total lysate, supernatant, and pulldown fractions by quantitative real-time PCR. Ago-IPs were performed in three independent biological replicates

Library generation, sequencing, and raw data processing

Library generation, quality control, and quantification:

Purified RNAs of the Ago, IgG and Input fractions were quantified using a Qubit fluorometer and the quality was assessed by an Agilent Bioanalyzer 2100 using RNA picoChips. 10 ng of RNA were treated with *DNase*I (Qiagen, Hilden, Germany). 8µl of Ago2-IP sample or 100pg of total RNA input material were utilized for cDNA library generation using 'NEBNext® Single Cell / Low Input RNA Library Prep Kit for Illumina®' (E6420S; New England Biolabs). All steps were performed as recommended in user manual E6420 (Version 1.0_04-2018; NEB) except that just half of the RT reaction products were used and accordingly all reactions were downscaled to 1/2 of initial volumes starting from cDNA amplification (point 2.4. of the manual). cDNA amplification was performed with 15 pcr cycles for input material and 17 cycles for RIP samples.

250pg of each amplified cDNA were used for fragmentation / end prep and adaptor ligation. After cleanup, processed cDNA samples were barcoded by single indexing approach, using 'NEBNext Multiplex Oligos for Illumina' (Index Primer A001-A013; A015-A021), applying 9 cycles of final pcr.

Fragment length distribution of amplified cDNA samples and individual libraries was monitored using 'Bioanalyzer High Sensitivity DNA Assay' (5067-4626; Agilent Technologies). Quantification of libraries was performed by use of the 'Qubit® dsDNA HS Assay Kit' (Q32854; ThermoFisher Scientific).

Library denaturation and Sequencing run:

Equal molar amounts of nineteen individually barcoded libraries were pooled. Accordingly, each analyzed library constitutes 5.3% of overall flowcell capacity. The library pool was denatured with NaOH and was finally diluted to 1.8pM according to the Denature and Dilute Libraries Guide (Document # 15048776 v02; Illumina). 1.3 ml of denatured pool was loaded on an Illumina NextSeq 550 sequencer using a High Output Flowcell for single reads (20024906; Illumina). Sequencing was performed with the following settings: Sequence read 1 with 76 bases; Index read 1 with 6 bases.

BCL to FASTQ conversion:

BCL files were converted to FASTQ files using bcl2fastq Conversion Software version v2.20.0.422 (Illumina).

Raw data processing and quality control:

Raw data processing was conducted by use of nfcore/rnaseq (version 1.3) which is a bioinformatics best-practice analysis pipeline used for RNA sequencing data at the National Genomics Infrastructure at SciLifeLab Stockholm, Sweden. The pipeline uses Nextflow, a bioinformatics workflow tool. It pre-processes raw data from FastQ inputs, aligns the reads and performs extensive quality-control on the results. The genome reference and annotation data were taken from GENCODE.org (Homo sapiens; GRCh38; release 29).

Normalization and relative RNA enrichment analysis:

Normalization was performed with DESeq2 (Galaxy Tool Version 2.11.40.2) with default settings except for "Output normalized counts table" which was set to "Yes". Relative RNA enrichment analysis and Wald statistics regarding the contrast C1 were also conducted using Deseq2. Resulting p-values were adjusted according to the approach recommended by Benjamini and Hochberg and used as an additional filter criterion (Fig. 1B).

Statistical modeling and analysis of Ago-RIP sequencing (Ago-RIP-Seq)

The identification of direct miR-449a-5p targets was achieved by using the concept of linear contrasts [12]. Following miR-449a-5p overexpression, the fraction of miR-449a-5p and its target genes is increased in the Ago complex. To reduce the noise due to unspecific RNA binding to the Protein G beads in the Ago-RIP-Seq experiment, the Ago-IP fractions were adjusted to IgG-IP [Contrast 1, C1] before comparing the Ago-IP fractions of miR-449a-5p with the Ago-IP of miR-control as follows:

[Contrast 1] = $(\text{Ago}_{\text{miR-449a}} - \text{IgG}_{\text{miR-449a}}) - (\text{Ago}_{\text{miR-Ctrl}} - \text{IgG}_{\text{miR-Ctrl}})$

Since miR-449a-5p overexpression leads to widespread secondary changes within the gene expression profiles that might impact the profiles of immunoprecipitation [12], it was necessary to adjust the levels of RNAs detected in the Ago-IP fractions to their expression levels measured in the total lysates (=Input) [Contrast 2, C2] as follows:

[Contrast 2] = $(\text{Ago}_{\text{miR-449a}} - \text{Input}_{\text{miR-449a}}) - (\text{Ago}_{\text{miR-Ctrl}} - \text{Input}_{\text{miR-Ctrl}})$

As microRNA targets are decreased after miR-449a-5p expression, the comparison [Contrast 3, C3] for identifying whole transcriptional changes was defined as follows:

[Contrast 3] = $(\text{Input}_{\text{miR-449a}} - \text{Input}_{\text{miR-Ctrl}})$

The contrasts C1, C2 and C3 define the type of comparison between factor levels. Wald-tests were applied for testing against zero.

Luciferase reporter assay

Potential miR-449a-5p binding sites in the 3'UTR of *PEA15, PPP1CA* and *TUFT1* were identified by TargetScan [2] or IntaRNA [3]. GBlocks^{TM} Gene Fragments (Integrated DNA Technologies) of 1916 bp (*PEA15*), 377 bp (*PPP1CA*) or 1902 bp (*TUFT1*) length were purchased containing wildtype or mutated miR-449a-5p binding sites, respectively. All gBlocks DNA fragments were

digested with *Xba*I and *Fse*I and cloned downstream of firefly luciferase into the pGL3-Promoter vector (Promega). For transfection, 8,000 HEK293T cells were seeded in white 96-well plates. After 24 hours combinations of 20 nM miRNA mimics (miR-Control or miR-449a-5p) and 25 ng luciferase reporter vectors (pGL3-Promoter, pGL3-wt, pGL3-mut) were transfected in triplicate using Lipofectamine 2000 (Life Technologies). For normalization, pGL4.70 (Promega) inserted with an EF1α promoter at the *Xho*I restriction site upstream of renilla luciferase, was co-transfected in all conditions. Luciferase activities were measured with the DualGlo Luciferase Assay System (Promega) 24 hours after transfection.

Tube formation assay

24-well plates were coated with 0.250 µL Matrigel Basement Membrane Matrix (BD Biosciences, Franklin Lakes, USA) and incubated at 37 °C. After 1 h, 5 x 104 HUVECs were seeded on Matrigel and co-cultured with an equivalent number of HLE cells in 500 µl EBM-2 medium. Co-cultured HLE cells were seeded in Transwell Permeable Supports (Corning, New York, USA) with a 0.4 µm polycarbonate membrane and incubated in the same well with HUVECs. The tube formation ability of HUVECs was imaged after 6 h of co-culture using an inverted light microscope. The number of tubes, segment length, junctions and nodes of the tubular structures was quantified by Angiogenesis Analyzer (ImageJ, [22]).

Taqman Assays for quantitative real-time PCR

**Target gene**	**Assay ID**	**Manufacturer**
CFL1	Hs00830568_g1	Life Technologies, Carlsbad, CA, USA
FOSL1	Hs00759776_s1	Life Technologies, Carlsbad, CA, USA
NDC1	Hs00917927_m1	Life Technologies, Carlsbad, CA, USA
PEA15	Hs00269428_m1	Life Technologies, Carlsbad, CA, USA
PPP1CA	Hs00267568_m1	Life Technologies, Carlsbad, CA, USA
TBP	Hs00920494_m1	Life Technologies, Carlsbad, CA, USA
TSPAN14	Hs01012920_m1	Life Technologies, Carlsbad, CA, USA
TUFT1	Hs01064705_m1	Life Technologies, Carlsbad, CA, USA

Antibodies for western blotting

Antigen	Dilution	Order no.	Manufacturer
α-actinin	1:3000	3134	Cell Signaling Technology, Danvers, USA
β-actin	1:2000	3700	Cell Signaling Technology, Danvers, USA
AKT	1:1000	9272	Cell Signaling Technology, Danvers, USA
ERK 1/2	1:1000	4695	Cell Signaling Technology, Danvers, USA
GAPDH	1:7500	Sc25778	Santa Cruz Biotechnology, Dallas, USA
PEA-15	1:1000	2780	Cell Signaling Technology, Danvers, USA
Phospho-AKT	1:1000	4060S	Cell Signaling Technology, Danvers, USA
Phospho-ERK 1/2	1:2000	4370	Cell Signaling Technology, Danvers, USA
PP1CA	1:1000	2582	Cell Signaling Technology, Danvers, USA
TUFT1	1:500	HPA028112	Sigma-Aldrich, St. Louis, USA

Antibodies for Ago2 RNA immunoprecipitation.

Antigen	Order no.	Manufacturer
Ago 2	11A9-100	Chromotek, Planegg-Martinsried, Germany
HA-tag (IgG)	7c9-100	Chromotek, Planegg-Martinsried, Germany

Buffer for Ago2 RNA immunoprecipitation:

1 x NP40 lysis buffer
50 mM Hepes-KOH (pH 7.5)
150 mM KCl
2 mM EDTA
1 mM NaF
0.05% Igepal
0.5 mM DTT
1 x EDTA-free Complete Protease Inhibitor Cocktail

Citrat phosphate buffer (pH 5)
25 mM citric acid
65 mM Na_2HPO_4

IP wash buffer (pH 7.5)
50 mM Hepes-KOH (pH 7.5)
300 mM KCl
0.05% Igepal
0.5 mM DTT
1 x EDTA-free Complete Protease Inhibitor Cocktail

High salt wash buffer (pH 7.5)

50 mM Hepes-KOH (pH 7.5)
500 mM KCl
0.05% Igepal
0.5 mM DTT
1 x EDTA-free Complete Protease Inhibitor Cocktail

Phosphatase wash buffer

50 mM Tris-HCl (pH 7.5)

20 mM EGTA

0.5% Igepal

PNK buffer

50 mM Tris-HCl (pH 7.5)

50 mM NaCl

10 mM $MgCl_2$

gblocks for cloning luciferase reporter vectors

(Overhang sequences to generate *Xba*I and *Fse*I restriction sites are indicated in italics. Predicted miR-449a-5p binding sites are underlined, mutated binding sites are indicated in bold. The gBlocks™ were purchased from Integrated DNA Technologies (IDT).

**gBlocks™ Gene Fragment**	**Sequence**
PEA15-3'UTR WT<br>(1890 bp)	*ATGATCTCTAGA*<br>GCAAGGGGGAGGAAGAGGAGGAAGGTTGGACCTTCATCAGACCACTCCCTTCCCC<br>CATCCTCCAGGAGAGGGGGCAAGGGCAACCCACCATCTACCCACTTACTAACCTGGTCCT<br>AACCCCCTTACTGTGCGCGTGTGTGTGCGTGTGCGCACGCTCTGGCTGTTTGTCTATATG<br>TCTAGCTCATCTAGTTCCTCTTCTTAAGGGGATGGGGGTCAGGGGCTAGGGGAGGGGGCT<br>GAGTTTCCCCACTTTAGGAGGAGGTGGGGGCTATTTCTATGCAAATAGAAATCAGCACAT<br>TCCTCCTACTTCCCTTTCCTCCACTCCCCCCATATCTTTAAAGTGTGGAAGCAGAAAGGA<br>CCTGCATTTTCCTACATTGAGGAGCTGACATAGGGGTAAGGTATGGGAGAGGTAGGTGGA<br>TCCAGGGAAAAGCAGTGGGGACGGAAGGCAAAGAGACCACTCAACCCCCACCTGGAAGGG<br>GCAAAGAAAAGCCAGAGTTCCATGTTTGTACTCCTGTGCTGGACTGTTTCCTGAGTACCA<br>GCAGGTCCCTTTTTGTCTCTCATGGGCCTAGCATAGGTATGAGCCAGGGATCCTTTCCTG<br>GTCCCTAAGATCAAACCCCATGGAGCAGCCAGCGTTAGATGCCCCCACCCACCTGTACTC<br>TGGAGAGACTGTGCTGGGAACATGTACCACTGAGCCTGAGATGGGGATGAGGGCAGAGAG<br>AGGGGAGCCCCCTCTTCCACTCAGTTGTTCCTACTCAGACTGTTGCACTCTAAACCTAGG<br>GAGGTTGAAGAATGAGACCCTTAGGTTTTAACACGAATCCTGACACCACCATCTATAGGG<br>TCCCAACTTGGTTATTGTAGGCAACCTTCCCTCTCTCCTTGGTGAAGAACATCCCAAGCC<br>AGAAAGAAGTTAACTACAGTGTTTTCCTTTGCACCGATCCCCACCCCAATTCAATCCCGG<br>AAGGGACTTACTTAGGAAACCCTTCTTTACTAGATATCCTGGCCCCCTGGGCTTGTGAAC<br>ACCTCCTAGCCACATCACTACAGTACAGTGAGTGACCCCAGCCTCCTGCCTACCCCAAGA<br>TGCCCCTCCCCACCCTGACCGTGCTAACTGTGTGTACATATATATTCTACATATATGTAT<br>ATTAAAACTGCACTGCCATGTCTGCCCTTTTTTGTGGTGTCTAGCATTAACTTATTGTCT<br>AGGCCAGAGCGGGGGTGGGAGGGGAATGCCACAGTGAAGGGAGTGGCAGAATCAAATTGC<br>TACATAGTCCAAACAAAAAAGAAGGCTTTTTCAAAAAACATTAAATTCACATGCAGTCTC<br>AGAGACTATTTAGACAAAGTTCAAGTTAGGAGCTTTTAGGATGTGGGAGTAAAACTTTAA

	TGGGAGGGGAGGGCTGGCTGCTGGAAGAAGGAAGAAGCCAGACTGGTTAGACAGTACTCT<br>TAACTCCTAGCCCAGCCTAGCGTGCCCTGCCCCTCTGGCCACTGCTGCAGACACCTGCCT<br>TAACACACACACCTCTAGGACTCCACAGTTTTGCCTTAAAGGACCTTCCCAAGTCTCCCT<br>TTCCCTGTCTGGCTTCTCCCTTAAGAAGAGAGAGATACTTGTAGAATTGGGTGGGGGGAA<br>TGAGCATGAACTGTCCTTCCATTTGGGATATGTTACATTAGAGTGAGAGAGAGAATAAGG<br>AGCCTTTCTTATGGAAGAAATGGGAGAAGAGAGACAGGGTTCTTTTCAGCAGAGTCTAGT<br>AGTTTCTCTGTAAGGCAAAATAATCTAAAAAGACTAACCTGCCCACCCACTCCTTATATT<br>GCTGTGAGATTGCCCCTATCTTGTGCTCTTCTGTCTGCAGTGTGCACGGCCTTGTTCTAA<br>CCCGGAATAAAGGTGATTGATTGTATTGCAACTAA *GGCCGGCCATGATC*
PEA15-3'UTR MUT<br><br>(1890 bp)	*ATGATCTCTAGA*<br>GCAAGGGGGAGGAAGAGGAGGAAGGTTGGACCTTCATCAGACCACTCCCTTCCCC<br>CATCCTCCAGGAGAGGGGGCAAGGGCAACCCACCATCTACCCACTTACTAACCTGGTCCT<br>AACCCCCTTACTGTGCGCGTGTGTGTGCGTGTGCGCACGCTCTGGCTGTTTGTCTATATG<br>TCTAGCTCATCTAGTTCCTCTTCTTAAGGGGATGGGGGTCAGGGGCTAGGGGAGGGGGCT<br>GAGTTTCCCCACTTTAGGAGGAGGTGGGGGCTATTTCTATGCAAATAGAAATCAGCACAT<br>TCCTCCTACTTCCCTTTCCTCCACTCCCCCCATATCTTTAAAGTGTGGAAGCAGAAAGGA<br>CCTGCATTTTCCTACATTGAGGAGCTGACATAGGGGTAAGGTATGGGAGAGGTAGGTGGA<br>TCCAGGGAAAAGCAGTGGGGACGGAAGGCAAAGAGACCACTCAACCCCCACCTGGAAGGG<br>GCAAAGAAAAGCCAGAGTTCCATGTTTGTACTCCTGTGCTGGACTGTTTCCTGAGTACCA<br>GCAGGTCCCTTTTTGTCTCTCATGGGCCTAGCATAGGTATGAGCCAGGGATCCTTTCCTG<br>GTCCCTAAGATCAAACCCCATGGAGCAGCCAGCGTTAGATGCCCCCACCCACCTGTACTC<br>TGGAGAGACTGTGCTGGGAACATGTACCACTGAGCCTGAGATGGGGATGAGGGCAGAGAG<br>AGGGGAGCCCCCTCTTCCACTCAGTTGTTCCTACTCAGACTGTTGCACTCTAAACCTAGG<br>GAGGTTGAAGAATGAGACCCTTAGGTTTTAACACGAATCCTGACACCACCATCTATAGGG<br>TCCCAACTTGGTTATTGTAGGCAACCTTCCCTCTCTCCTTGGTGAAGAACATCCCAAGCC<br>AGAAAGA**CTGGCCATCACTGTGG**TTCCTTTGCACCGATCCCCACCCCAATTCAATCCCGG<br>AAGGGACTTACTTAGGAAACCCTTCTTTACTAGATATCCTGGCCCCCTGGGCTTGTGAAC<br>ACCTCCTAGCCACATCACTACAGTACAGTGAGTGACCCCAGCCTCCTGCCTACCCCAAGA<br>TGCCCCTCCCCACCCTGACCGTGCTAACTGTGTGTACATATATATTCTACATATATGTAT<br>ATTAAAAC**GTCAAGTAAC**TGTCTGCCCTTTTTTGTGGTGTCTAGCATTAACTTATTGTCT<br>AGGCCAGAGCGGGGGTGGGAGGGGAATGCCACAGTGAAGGGAGTGGCAGAATCAAATTGC<br>TACATAGTCCAAACAAAAAAGAAGGCTTTTTCAAAAAACATTAAATTCACATGCAGTCTC<br>AGAGACTATTTAGACAAAGTTCAAGTTAGGAGCTTTTAGGATGTGGGAGTAAAACTTTAA<br>TGGGAGGGGAGGGCTGGCTGCTGGAAGAAGGAAGAAGCCAGACTGGTTAGACAGTACTCT<br>TAACTCCTAGCCCAGCCTAGCGTGCCCTGCC**AAGAGTTGACTGTAG**GCAGACACCTGCCT<br>TAACACACACACCTCTAGGACTCCACAGTTTTGCCTTAAAGGACCTTCCCAAGTCTCCCT

	TTCCCTGTCTGGCTTCTCCCTTAAGAAGAGAGAGATACTTGTAGAATTGGGTGGGGGGAA<br>TGAGCATGAACTGTCCTTCCATTTGGGATATGTTACATTAGAGTGAGAGAGAGAATAAGG<br>AGCCTTTCTTATGGAAGAAATGGGAGAAGAGAGACAGGGTTCTTTTCAGCAGAGTCTAGT<br>AGTTTCTCTGTAAGGCAAAATAATCTAAAAAGACTAACCTGCCCACCCACTCCTTATATT<br>GCTGTGAGATTGCCCCTATCTTGTGCTCTTCTGTCTGCAGTGTGCACGGCCTTGTTCTAA<br>CCCGGAATAAAGGTGATTGATTGTATTGCAACTAA *GGCCGGCCATGATC*
PPP1CA-3'UTR WT<br>(352 bp)	*GATATATCTAGA*<br>CCCCCGCACACCACCCTGTGCCCCAGATGATGGATTGATTGTACAGAAATCATGCTGCCA<br>TGCTGGGGGGGGGTCACCCCGACCCCTCAGGCCCACCTGTCACGGGGAACATGGAGCCTT<br>GGTGTATTTTTCTTTTCTTTTTTTAATGAATCAATAGCAGCGTCCAGTCCCCCAGGGCTG<br>CTTCCTGCCTGCACCTGCGGTGACTGTGAGCAGGATCCTGGGGCCGAGGCTGCAGCTCAG<br>GGCAACGGCAGGCCAGGTCGTGGGTCTCCAGCCGTGCTTGGCCTCAGGGCTGGCAGCCGG<br>ATCCTGGGGCAACCCATCTGGTCTCTTGAATAAAGGTCAAAGCTGGATTCTC<br>*GGCCGGCCGATAGT*
PPP1CA-3'UTR<br>MUT<br>(352 bp)	*ATGATCTCTAGA*<br>CCCCCGCACACCACCCTGTGCCCCAGATGATGGATTGATTGTACAGAAATC**C**T**T**C**G**G**A**C**C**<br>TGCTGGGGGGGGGTCACCCCGACCCCTCAGGCCCACCTGTCACGGGGAACATGGAGCCTT<br>GGTGTATTTTTCTTTTCTTTTTTTAATGAATCAATAGCAGCGTCCAGTCCCCCAGGGCTG<br>CTTCCTGCCTGCACCTGCGGTGACTGTGAGCAGGATCCTGGGGCCGAGGCTGCAGCTCAG<br>GGCAACGGCAGGCCAGGTCGTGGGTCTCCAGCCGTGCTTGGCCTCAGG**T**C**G**G**T**C**C**GCCGG<br>ATCCTGGGGCAACCCATCTGGTCTCTTGAATAAAGGTCAAAGCTGGATTCTC<br>*GGCCGGCCATGATC*
TUFT1-3'UTR WT<br>(1876 bp)	*ATGATCTCTAGA*<br>GCTGCCTGGAGATGGTTGCTGCCATTGCTGCTGCCTCTGCCTCGGAGAAGCCCACT<br>GCCCCTGTTGGCTGTTAACACTGCCTTTGACTTCCTGACTGTCCCCTGGCTGCACCCAGG<br>ACTTCGGGCTCCTGTGTCTCACCATTCCCAAGCCCCTGGCCACTCTAAGCTGGGCAGACG<br>GAGCACGAGCACCTATTCAAGGCACTGCAGCCCTTTGGAAGACATTGTCCTGCAAGCAGG<br>AGCCAGGGCAATATCTATATTCCTACAGTGACTATTTTTCTCTGTAGAGAGCCTCCCTTC<br>TGTTGTAGACTGGACTCTGGCTGTGCCATAAGCCAGGCCTTCATCAGATTGGGAGAGGTG<br>ACAAGATTTGCCTCAGCCCTAAAAGCTGGAGACACAGATGTCCAGAGTGATTGGAGAATG<br>TCCTGGGGGAATGAAGTTCCTTCCACAAACACAGCTCAGTTCTTAGCAACAAACTGTTTG<br>TTTTTCTACTTGCTCCATCTGCAGCCTACGCTGCCCTGGCCTCCTGCAGACAGATAGTGG<br>GGTTACCTGGCAAGGCCTGGTGAGAGCCAGTGAACCTAAGCTTTGACTGGGTGGCCTTGT<br>CTTTCTGGGGAGGAGGGAATGTACATTCAGGGAGTAGCCTTTTGCGGAAAAATTCTCTAG<br>GGCTACAGACAGTCATGTGTGACTTCTCTCTGCTGTGAAAACTCCCAGAGTCTCTTTAGG<br>GATTTTCCCTAAGGTGTACCACCAGGCACACCTCAGTCTTCTTGACCCAGAGCCTGAAAA

	CTGTTTTCACTGGGTTCCACCAGTCCCAGCAAAATCCTCTTTGTATTTATTTTGCTAAGT<br>TATTGGTGGTTTTGCTTACATCTCATGATTGATATAATACCAAAGTTCTATAGCCTTCTC<br>TTGCAGTATTTGGATTTGCTTGAAACCGGGAAAACTGTTCCCATTAGGCTTGTTAATGTC<br>AGAGTGACACTATTATGAATCTTTCTCTCCCTTTCCTCTGCCTGTTTCTTCTCTCTTTCT<br>CCTTCAAACTTGCTCTGCAGCTAAGGAAGGTGAGTCTACTTTCCCTGAGGCTTTGGGGTC<br>AGAGTATATGTTGTTTGGAGAAAGAGGGCAATCAGGACTCTTCTGGGACCCAGATGAGTT<br>CTTCACTAGCCCTTCTGAACCCCTTGCTCCATAATTGGTCTTTTATCCTGGCTCTGAATG<br>ACCCTGCAGGTCATCATGGTTTTCTTTTTTTATTGTTTTTTTTCTTTTCTGAGACAGAGT<br>CTCACTCTGTCACCCAGGCTGGAGTGCAGTGGCGCGATCTCAGCTCACTGCAACCTCTGC<br>CTCCCGGATTTAAGCGATTCTTCTGCCTCAGCCTCCCGAGTAGCTGGGACTACAGGTGTG<br>CCACCACGCCTGGCTGATTTTTGTATTTTTAGTAGAGATGGGGTTTCACCATACTGGCTA<br>GGCTGGTCTCGAATTCCTGACCTCAGGTGATCCACCCACCTCGGCTTCCCAAAGTGCTAG<br>GATTATAGGCTTGAGCTACTGCGCCCGGCCCATGGTGTTTTTCTTTAGGGCTCTTCCTAC<br>AACCTTGAGAAGTAGATAGGCATCAGAGTATGGTACTATAGGAATCAGAAAAATTCAAAA<br>CAAATGTGGATTAAGTGTTTAGGCTCTATGTGGCTCACGCAGCCAGAATCCTTAAGTCTG<br>TGTGTTTCTGTGTCTCAAGACTGGGCTCACATTCTGGCTTTGTCCATAACAATGCTCTGG<br>GATTTCAGGGAGTTCCCTCATTTGTAAAATGAGGGGGTCAGAGCAGGTGATATCCATGTT<br>TCTTCCCTTTCTGATATTGTTGTCTGTGGCATATTCTTTGTATGGCGAATTTAATAAATT<br>ATATTAATGTGTCTCTTTGA<br>*GGCCGGCCATGATC*
TUFT1-3’UTR MUT<br><br>(1876 bp)	*ATGATCTCTAGA*<br>GCTGCCTGGAGATGGTTGCTGCCATTGCTGCTGCCTCTGCCTCGGAGAAGCC**A**A**A**T<br>**T**C**AA**C**G**G**G**T**T**G**A**TGTTA**C**C**T**C**G**G**A**CTTTGACTTCCTGACTGTCCCCTGGCTGCACCCAGG<br>ACTTCGGGCTCCTGTGTCTCACCATTCCCAAGCCCCTGGCCACTCTAAGCTGGGCAGACG<br>GAGCACGAGCACCTATTCAAGGCACTGCAGCCCTTTGGAAGACATTGTCCTGCAAGCAGG<br>AGCCAGGGCAATATCTATATTCCTACAGTGACTATTTTTCTCTGTAGAGAGCCTCCCTTC<br>TGTTGTAGACTGGACTCTGGCTGTGCCATAAGCCAGGCCTTCATCAGATTGGGAGAGGTG<br>ACAAGATTTGCCTCAGCCCTAAAAGCTGGAGACACAGATGTCCAGAGTGATTGGAGAATG<br>TCCTGGGGGAATGAAGTTCCTTCCACAAACACAGCTCAGTTCTTAGCAACAAACTGTTTG<br>TTTTTCTACTTGCTCCATCTGCAGCC**G**A**A**G**A**T**T**C**A**CTGGCCTCCTGCAGACAGATAGTGG<br>GGTTACCTGGCAAGGCCTGGTGAGAGCCAGTGAACCTAAGCTTTGACTGGGTGGCCTTGT<br>CTTTCTGGGGAGGAGGGAATGTACATTCAGGGAGTAGCCTTTTGCGGAAAAATTCTCTAG<br>GGCTACAGACAGTCATGTGTGACTTCTCTCTGCTGTGAAAACTCCCAGAGTCTCTTTAGG<br>GATTTTCCCTAAGGTGTACCACCAGGCACACCTCAGTCTTCTTGACCCAGAGCCTGAAAA<br>CTGTTTTCACTGGGTTCCACCAGTCCCAGCAAAATCCTCTTTGTATTTATTTTGCTAAGT<br>TATTGGTGGTTTTGCTTACATCTCATGATTGATATAATACCAAAGTTCTATAGCCTTCTC

	TTGCAGTATTTGGATTTGCTTGAAACCGGGAAAACTGTTCCCATTAGGCTTGTTAATGTC<br>AGAGTGACACTATTATGAATCTTTCTCTCCCTTTCCTCTGCCTGTTTCTTCTCTCTTTCT<br>CCTTCAAACTTGCTCTGCAGCTAAGGAAGGTGAGTCTACTTTCCCTGAGGCTTTGGGGTC<br>AGAGTATATGTTGTTTGGAGAAAGAGGGCAATCAGGACTCTTCTGGGACCCAGATGAGTT<br>CTTCACTAGCCCTTCTGAACCCCTTGCTCCATAATTGGTCTTTTATCCTGGCTCTGAATG<br>ACCCTGCAGGTCATCATGGTTTTCTTTTTTTATTGTTTTTTTTTTTTTCTGAGACAGAGT<br>CTCACTCTGTCACCCAGGCTGGAGTGCAGTGGCGCGATCTCAGCTCACTGCAACCTCTGC<br>CTCCCGGATTTAAGCGATTCTTCTGCCTCAGCCTCCCGAGTAGCTGGGACTACAGGTGTG<br>CCACCACGCCTGGCTGATTTTTGTATTTTTAGTAGAGATGGGGTTTCACCAT**C**C**G**G**T**C**G**A<br>GGCTGGTCTCGAATTCCTGACCTCAGGTGATCCACCCACCTCGGCTTCCCAAAGTGCTAG<br>GATTATAGGCTTGAGCTACTGCGCCCGGCCCATGGTGTTTTTCTTTAGGGCTCTTCCTAC<br>AACCTTGAGAAGTAGATAGGCATCAGAGTATGGTACTATAGGAATCAGAAAAATTCAAAA<br>CAAATGTGGATTAAGTGTTTAGGCTCTATGTGGCTCACGCAGCCAGAATCCTTAAGTCTG<br>TGTGTTTCTGTGTCTCAAGACTGGGCTCACATTCTGGCTTTGTCCATAACAATGCTCTGG<br>GATTTCAGGGAGTTCCCTCATTTGTAAAATGAGGGGGTCAGAGCAGGTGATATCCATGTT<br>TCTTCCCTTTCTGATATTGTTGTCTGTGGCATATTCTTTGTATGGCGAATTTAATAAATT<br>ATATTAATGTGTCTCTTTGA<br>*GGCCGGCCATGATC*

Supplementary Figures

Suppl. Fig. 1. siPOOL establishment of siPEA15, siPPP1CA and siTUFT1

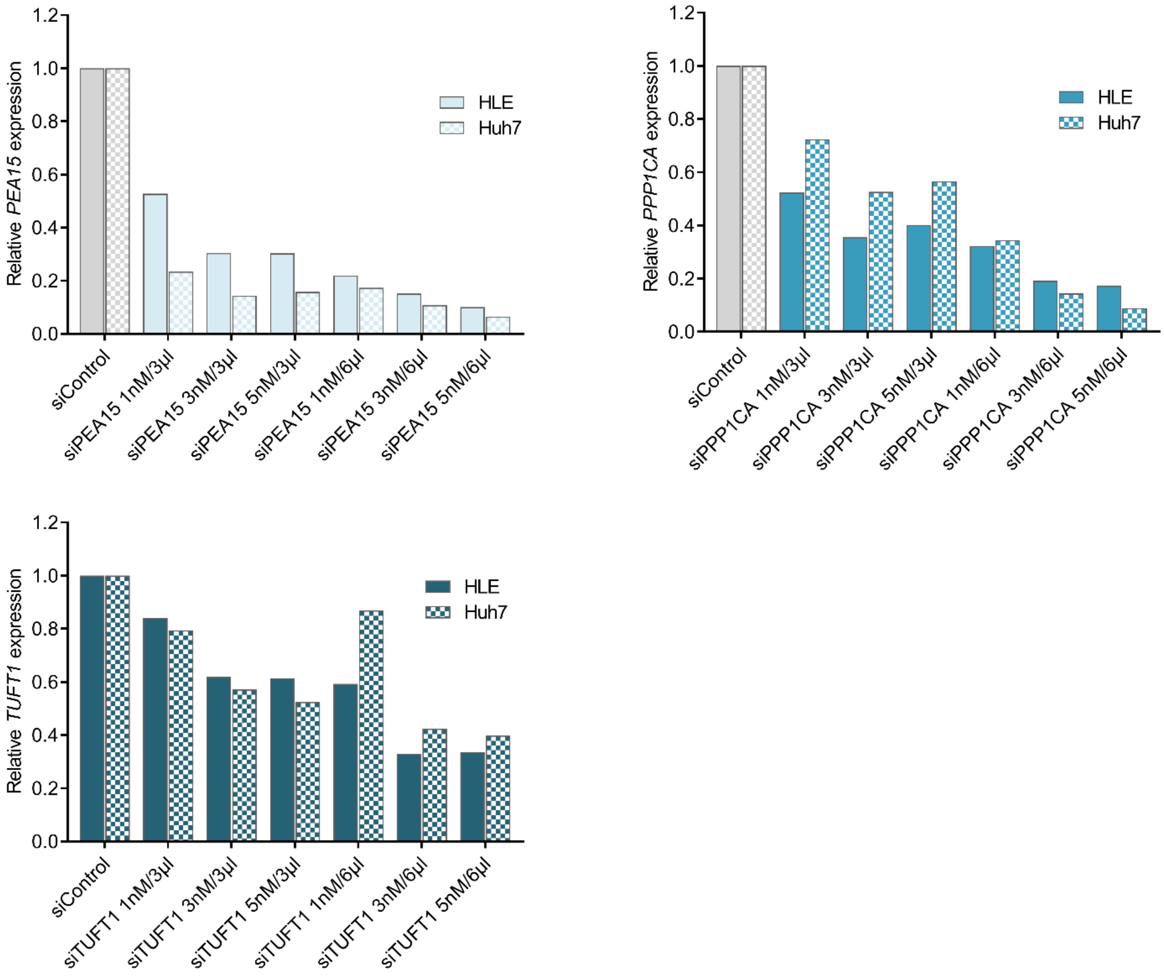

Suppl. Fig. 1. siPOOL establishment of siPEA15, siPPP1CA and siTUFT1

Different siPOOL (siTOOLs Biotech) concentrations were used and siPOOLs were transfected with either 3 µl or 6 µl HiPerFect Transfection Reagent to establish the best condition for RNA knockdown.

Suppl. Fig. 2. MiR-449a and knockdown of *PEA15, PPP1CA* and *TUFT1* increase sorafenib efficacy in HCC

A

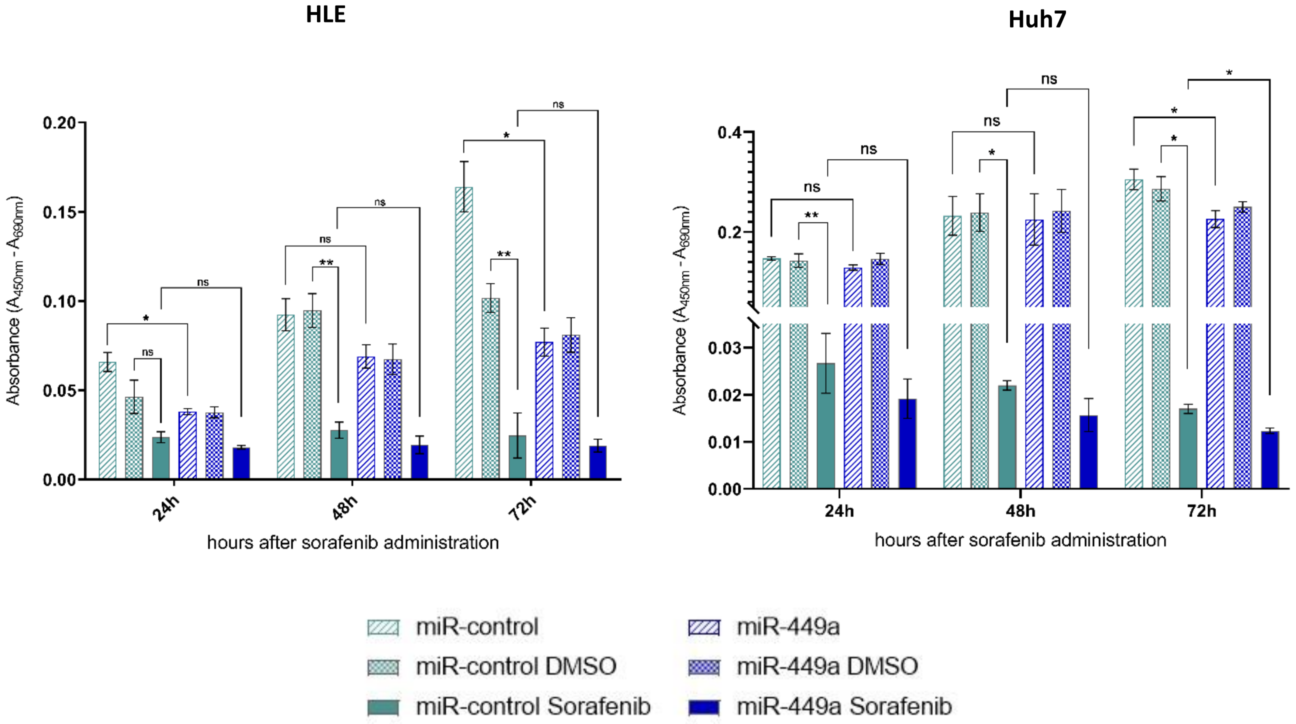

B

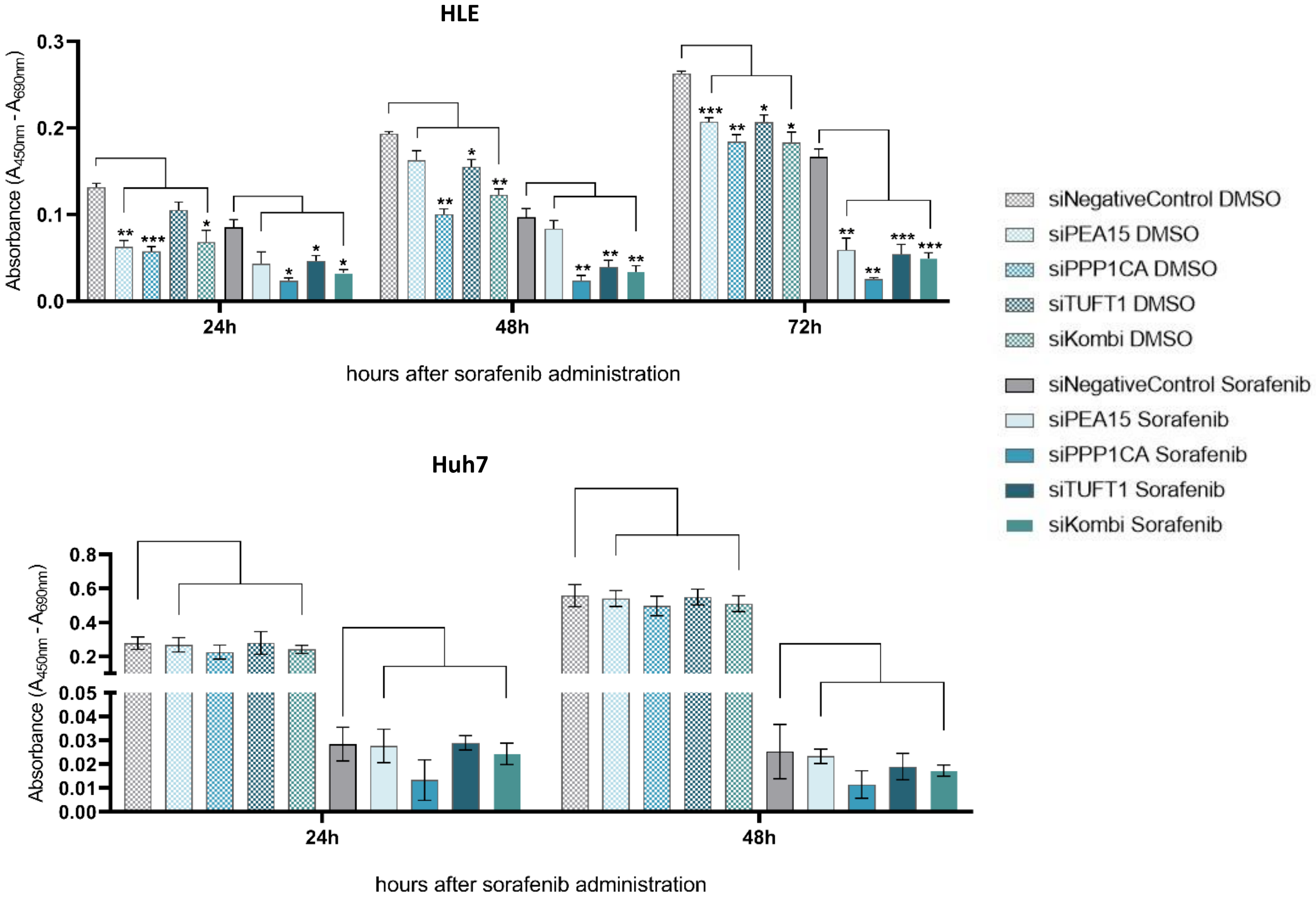

Suppl. Fig. 2. MiR-449a-5p and knockdown of *PEA15, PPP1CA* and *TUFT1* increase sorafenib efficacy in HCC

(A) HLE and Huh7 cells were transfected with miR-449a-5p mimic/ miR-control and treated with 10 µM sorafenib or DMSO vehicle control. 24 h, 48 h and 72 h after sorafenib treatment cell viability was measured; two-way ANOVA with Tukey's multiple comparisons test (square brackets). (B) HLE and Huh7 cells were transfected with siRNA pools, either alone or in combination (siCombi) and treated with 10 µM sorafenib or DMSO vehicle control. 24 h, 48 h and 72 h after sorafenib treatment cell viability was measured; *p<0.05, **p<0.01, ***p<0.001, ****p<0.0001; two-way ANOVA with Dunnett's multiple comparisons test (square brackets).

Supplementary Tables

Supplementary Table 1. Potential miR-449a-5p target genes identified by Ago-RIP-Seq

Nr.	Gene name				
1	EIF3H	43	ZC3H15	85	CAV1
2	PPARG	44	PEA15	86	HMGN2P5
3	CDC26	45	CCND1	87	TGFBI
4	IRF2BPL	46	KAT2A	88	HMGN2P3
5	SOX4	47	IDH3B	89	AL139095.2
6	RDH11	48	TK1	90	TUT1
7	HMMR	49	TPD52	91	STRAP
8	FTO	50	SCAMP3	92	DMAC1
9	VAMP2	51	MDH2	93	C2orf74
10	MDM4	52	GAPDH	94	SNX19
11	CYREN	53	PHB	95	C3orf52
12	FAM76A	54	LDHA	96	ARSJ
13	LMAN2L	55	HSPA1A	97	BEX1
14	TPM2	56	HIF1A	98	CCDC85B
15	TUFT1	57	TUFM	99	TMA7
16	LGR4	58	GLB1	100	RCC2
17	PEX11B	59	BCKDK	101	NR3C1
18	LINC00667	60	USP11	102	HMGN2P17
19	SHMT2	61	KIAA1191	103	STIM1
20	SEC23A	62	MRTO4	104	MRPL11
21	TSPAN14	63	RALBP1	105	FAM204A
22	LPIN3	64	FOSL1	106	E2F3
23	RNF182	65	HGS	107	MPST
24	AP1S1	66	PGP	108	ASNA1
25	PANK2	67	AL031729.1	109	BOD1L1
26	RPS3	68	SOCS7	110	PAFAH2
27	ARL6IP1	69	PRMT1	111	KRT18P28
28	CHMP1B	70	AC027309.2	112	SPRYD4
29	NCSTN	71	NOL8	113	TBCB
30	DBN1	72	EFNA1	114	RBBP4P1
31	LRRC40	73	HMGCS1	115	RBM23
32	PPP1CA	74	Z82206.1	116	CLASP2
33	SLC25A39	75	RPL3	117	AL356000.1
34	PNO1	76	PPP4R2	118	ARMC8
35	MCM5	77	NBL1	119	PMF1
36	PPIA	78	RPL3P4	120	PPIAP22
37	GTF3C6	79	GLTP	121	DTYMK
38	CSNK2A1	80	ECPAS	122	AXL
39	TROAP	81	DRAP1	123	RPN2
40	CFL1	82	FDFT1	124	MTFR1L
41	NDC1	83	RPLP0	125	MPLKIP
42	UBE2I	84	HMGN2	126	HK1

127	ERLIN1	164	CORO1B
128	HS2ST1	165	FANCG
129	PMS2P1	166	TAGLN
130	ZNF706	167	LRR1
131	MCM3	168	PRR14
132	PRPF39	169	HMBS
133	AC011481.1	170	LDHAP4
134	MAIP1	171	CKAP2L
135	BRD4	172	PAPOLG
136	EPB41L4A-AS1	173	CLPB
137	SIRT1	174	LYRM4
138	MYOCD	175	PRKAR1B
139	LDHAP2	176	KRT8P3
140	IFRD2	177	TPRKB
141	MMAA	178	CHCHD2
142	MRPL19	179	DDX10
143	TRIM28	180	SEMA3E
144	DSN1	181	PPIAP29
145	SNHG5	182	ARHGDIA
146	FKBPL		
147	CIZ1		
148	SLC12A2		
149	LDHAP5		
150	ARL6IP5		
151	TM7SF3		
152	EDIL3		
153	PHF6		
154	GRIPAP1		
155	B3GNTL1		
156	ASNSD1		
157	KRI1		
158	TUBB		
159	AP2A2		
160	C19orf24		
161	LASP1		
162	VPS52		
163	CDCA5		

MiR-449a-5p target genes were defined by positive log2FC values in contrast 1 and contrast2 and negative log2FC values in lysate (contrast 3). Therefore, Ago-RIP-Sequencing yielded 182 potential direct miR-449a-5p target genes that fulfilled those conditions in all three replicates.

References

[1] Quintavalle C, Hindupur SK, Quagliata L, Pallante P, Nigro C, Condorelli G, et al. Phosphoprotein enriched in diabetes (PED/PEA15) promotes migration in hepatocellular carcinoma and confers resistance to sorafenib. Cell Death Dis 2017;8:e3138–e3138. https://doi.org/10.1038/cddis.2017.512.

[2] Agarwal V, Bell GW, Nam J-W, Bartel DP. Predicting effective microRNA target sites in mammalian mRNAs. ELife 2015;4:e05005. https://doi.org/10.7554/eLife.05005.

[3] Mann M, Wright PR, Backofen R. IntaRNA 2.0: enhanced and customizable prediction of RNA–RNA interactions. Nucleic Acids Research 2017;45:W435–9. https://doi.org/10.1093/nar/gkx279.
